# Deep5hmC: predicting genome-wide 5-hydroxymethylcytosine landscape via a multimodal deep learning model

**DOI:** 10.1093/bioinformatics/btae528

**Published:** 2024-08-28

**Authors:** Xin Ma, Sai Ritesh Thela, Fengdi Zhao, Bing Yao, Zhexing Wen, Peng Jin, Jinying Zhao, Li Chen

**Affiliations:** Department of Biostatistics, University of Florida, Gainesville, FL 32603, United States; Department of Biostatistics, University of Florida, Gainesville, FL 32603, United States; Department of Biostatistics, University of Florida, Gainesville, FL 32603, United States; Department of Human Genetics, Emory University School of Medicine, Atlanta, GA 30322, United States; Department of Psychiatry and Behavioral Sciences, Emory University School of Medicine, Atlanta, GA 30322, United States; Department of Human Genetics, Emory University School of Medicine, Atlanta, GA 30322, United States; Department of Epidemiology, University of Florida, Gainesville, FL 32603, United States; Department of Biostatistics, University of Florida, Gainesville, FL 32603, United States

## Abstract

**Motivation:**

5-Hydroxymethylcytosine (5hmC), a crucial epigenetic mark with a significant role in regulating tissue-specific gene expression, is essential for understanding the dynamic functions of the human genome. Despite its importance, predicting 5hmC modification across the genome remains a challenging task, especially when considering the complex interplay between DNA sequences and various epigenetic factors such as histone modifications and chromatin accessibility.

**Results:**

Using tissue-specific 5hmC sequencing data, we introduce Deep5hmC, a multimodal deep learning framework that integrates both the DNA sequence and epigenetic features such as histone modification and chromatin accessibility to predict genome-wide 5hmC modification. The multimodal design of Deep5hmC demonstrates remarkable improvement in predicting both qualitative and quantitative 5hmC modification compared to unimodal versions of Deep5hmC and state-of-the-art machine learning methods. This improvement is demonstrated through benchmarking on a comprehensive set of 5hmC sequencing data collected at four developmental stages during forebrain organoid development and across 17 human tissues. Compared to DeepSEA and random forest, Deep5hmC achieves close to 4% and 17% improvement of Area Under the Receiver Operating Characteristic (AUROC) across four forebrain developmental stages, and 6% and 27% across 17 human tissues for predicting binary 5hmC modification sites; and 8% and 22% improvement of Spearman correlation coefficient across four forebrain developmental stages, and 17% and 30% across 17 human tissues for predicting continuous 5hmC modification. Notably, Deep5hmC showcases its practical utility by accurately predicting gene expression and identifying differentially hydroxymethylated regions (DhMRs) in a case–control study of Alzheimer’s disease (AD). Deep5hmC significantly improves our understanding of tissue-specific gene regulation and facilitates the development of new biomarkers for complex diseases.

**Availability and implementation:**

Deep5hmC is available via https://github.com/lichen-lab/Deep5hmC

## 1 Introduction

5-Hydroxymethylcytosine (5hmC) modification is one important intermediate state among a succession of states in active demethylation, which includes 5-methylcytosine (5mC), 5hmC, 5-formylcytosine (5fC), and 5-carboxylcytosine (5caC). The generation of 5hmC occurs through the oxidation of 5mC by the ten-eleven translocation (TET) protein family, and it is specifically recognized by 5hmC-binding proteins (Tahiliani *et al.* 2009, Spruijt *et al.* 2013). In the nervous system, 5hmC plays a critical role in neurodevelopment and neurological function. It has been found to be enriched in embryonic stem cells and neuronal cells, regulating neuronal-specific gene expression during neural progenitor cell differentiation (Kriaucionis and Heintz 2009, Li *et al.* 2017b). Abnormalities in 5hmC distribution and enrichment can be critical factors contributing to neurodegenerative diseases such as Huntington’s disease, Autism spectrum disorder, and Alzheimer’s disease (AD) (Wang *et al.* 2013, Coppieters *et al.* 2014, Bernstein *et al.* 2016, Cheng *et al.* 2018, Qin *et al.* 2020, Kuehner *et al.* 2021). Beyond neurodegenerative diseases, 5hmC also plays a significant role in cancer development and treatment. Genome-wide mapping of 5hmC reveals that loss of 5hmC is an epigenetic hallmark of melanoma and medulloblastoma (Lian *et al.* 2012, Stahl *et al.* 2021, Zhao *et al.* 2021). Additionally, 5hmC in circulating cell-free DNA (cfDNA) serves as a diagnostic biomarker for colorectal, gastric, pancreatic cancer, acute myeloid leukemia, and other common human cancer types (Li *et al.* 2017, Gao *et al.* 2019, Shao *et al.* 2022). Importantly, 5hmC-based biomarkers of circulating cfDNA have demonstrated high predictiveness of cancer stage and are superior to conventional biomarkers (Li *et al.* 2017, Song *et al.* 2017, Guler *et al.* 2020).

The emergence of next-generation sequencing has facilitated the genome-wide profiling of 5hmc modification. Among 5hmC sequencing technologies, antibody-based immunoprecipitation and sequencing of hydroxymethylated DNA (hMeDIP-seq) (Weber *et al.* 2005), as well as 5hmC-selective chemical labeling method (e.g. 5hmC-Seal) (Song *et al.* 2011, Han *et al.* 2016), have become cost-effective methods to map genome-wide 5hmC signals. These methods aim to capture and enrich 5hmC methylated DNA fragments, followed by next-generation sequencing. Similar to ChIP-seq analysis, the enriched region of 5hmC, deemed a “peak,” can be identified by a peak-calling algorithm such as MACS (Zhang *et al.* 2008). Due to their popularity, hMeDIP-seq/5hmC-Seal and other similar protocols have been widely adopted to explore the distribution and patterns of genome-wide 5hmC in various tissues, cell types (Li *et al.* 2017, Cui *et al.* 2020) and diseases (Bernstein *et al.* 2016, Cheng *et al.* 2018, Qin *et al.* 2020, Kuehner *et al.* 2021). In addition, 5hmC sequencing aids in investigating the association between 5hmC and other genomic elements. For instance, 5hmC has been found to co-localize with gene bodies and enhancers known to activate gene expression, and it is positively correlated with gene expression (He *et al.* 2021). Moreover, 5hmC is significantly enriched in histone modifications associated with active enhancers, such as H3K4me1 and H3K27ac (Stroud *et al.* 2011, Cui *et al.* 2020). Furthermore, the distribution and enrichment of 5hmC exhibit tissue-specificity, evident in its preferential enrichment on tissue-specific gene bodies and enhancers (Li and Liu 2011). Cell type-specific 5hmC profiles have been found associated with transcriptional abundance and chromatin accessibility across human hematopoiesis (Nakauchi *et al.* 2022).

Nevertheless, it is still costly to conduct deep sequencing for an accurate identification of 5hmC modification. In addition, 5hmC experiments may suffer from underpowering due to insufficient sequencing depth or various artifacts, leading to a limited detection of 5hmC modification sites. Moreover, 5hmC profiles exhibit dynamic changes across tissues and cell types. To overcome these challenges, computational models have been developed to enable an *in silico* genome-wide prediction of 5hmC profiles. The general principle of these methods is to treat DNA sequence within a genomic region as the model input and predict the probability of the region being a 5hmC peak. The key distinction between these approaches lies in the feature engineering applied to DNA sequences and the successive use of machine learning algorithms. For example, iRNA5hmC-PS adopts *k*-mer (*k* = 2,3) frequency and uses logistic regression for predicting the presence of 5hmC peaks (Ahmed *et al.* 2020). iRhm5CNN utilizes a simple convolution neural network, which employs one-hot encoding DNA sequence as the model input to predict the occurrence of 5hmC peaks (Ali *et al.* 2021). Given that the resolution of 5hmC peaks closely aligned with that of ChIP-seq peaks, deep learning methods designed for predicting the binding sites of transcriptional factor, histone modification sites, and open chromatin regions such as DeepSEA (Zhou and Troyanskaya 2015), which aims to predict epigenetic signals across hundreds of tissues and cell types in a multi-task framework, can be readily adapted for the task for predicting 5hmC peaks.

Despite the success of existing computational methods for predicting 5hmC modification, there are still challenges to be addressed. First, there is a lack of computational methods specifically designed for predicting tissue- or cell-type-specific DNA 5hmC modification, and alternative methods used for this purpose, such as DeepDEA, may be suboptimal. Second, the relationship among 5hmC, other epigenetic features such as histone modification and chromatin accessibility, and gene expression is rarely explored in the predictive modeling of 5hmC modification. Lastly, current methods primarily concentrate on classifying binary 5hmC peaks while overlooking the quantitative variation of 5hmC modification. To address these challenges, we introduce a novel multi-modal deep learning framework named Deep5hmC, which aims to enhance the prediction of tissue/cell type-specific genome-wide 5hmC modification by incorporating information from both DNA sequence and epigenetic features such as histone modification and open chromatin accessibility. The contribution of our work lies on the following aspects: (i) Deep5hmC leverages both DNA sequence and other epigenetic features such as histone modification and chromatin accessibility to improve the prediction of 5hmC modification in both qualitative (i.e. 5hmC peaks) and quantitative prediction (i.e. normalized 5hmC reads); (ii) Deep5hmC is developed and evaluated using a comprehensive set of 5hmC sequencing (5hmc-seq) data collected at four developmental stages during forebrain organoid development and across 17 human tissues. The extensive dataset demonstrates the power of Deep5hmC in predicting tissue-specific 5hmC modification; (iii) Deep5hmC is further assessed using one 5hmC-seq data in one case–control study of AD to demonstrate its broad utility in predicting differentially hydroxymethylated regions (DhMRs) within the context of the disease; (iv) an extension of Deep5hmC is to quantify gene expression by predicted quantitative 5hmC modification within gene bodies; and (v) Deep5hmC is released as an open-source python toolkit, which can benefit the epigenetic research community. As a result, we demonstrate that the inclusion of histone modification or chromatin accessibility leads to improved prediction performance of Deep5hmC for both qualitative and quantitative 5hmC modification. Importantly, Deep5hmC outperforms competing machine learning approaches for the same purpose. In addition, Deep5hmC achieves an accurate prediction of gene expression and is also powerful for predicting DhMRs.

## 2 Materials and methods

### 2.1 Data description and processing

The first dataset, termed “Forebrain Organoid,” includes paired 5hmC-seq data and RNA-seq data across the embryoid body (8 days EB) and forebrain organoids cultured over three distinct developmental stages: 56 (D56), 84 (D84), and 112 days (D112), designed to model the early development of the fetal brain (Kuehner *et al.* 2021). The called 5hmC peaks using MACS2 are retrieved from the original publication with GEO accession number GSE151818 (Kuehner *et al.* 2021). Each 5hmC peak is subsequently standardized into a 1 kb window by extending the center of the peak upstream and downstream 500 bp. For the acquisition of raw read counts associated with each peak, the raw 5hmC-seq data are downloaded, and bowtie2 (Langmead *et al.* 2009) is employed to map the reads onto the hg19 reference genome. Using the R/Bioconductor package “GenomicRanges,” read counts for each 5hmC peak are obtained by overlapping the genomic positions of reads and peaks. Similarly, raw RNA-seq data are obtained, and STAR (Dobin and Gingeras 2015) is utilized to map the reads onto the hg19 reference transcriptome. Read counts for each gene are calculated based on the positional overlap between reads and genes, using the R/Bioconductor package “GenomicRanges” and “Rsamtools.” Subsequently, the read counts of biological replicates are averaged after adjusting the sequencing depth.

The second dataset, referred to as “Human Tissues,” comprises paired 5hmC-seq data and RNA-seq data spanning 19 human tissues derived from ten organ systems. The called 5hmC peaks using MACS2 are obtained from the original publication with GEO accession number GSE144530 (Cui *et al.* 2020). To enhance reliability, we merge the peaks from biological replicates and retain only those peaks appearing in more than two biological replicates. Subsequently, the merged peaks are further standardized into 1 kb windows. Raw 5hmC-seq data are downloaded and processed by mapping reads onto the hg19 reference genome using bowtie2. Read counts for each 5hmC peak are then calculated based on the mapped genomic positions. Raw RNA-seq data are downloaded and processed using STAR to map reads onto the hg19 reference transcriptome. The read counts for each gene are determined by overlapping genomic positions between reads and genes. The read counts of biological replicates are averaged while adjusting the sequencing depth.

The third dataset, titled “Kentucky AD,” is obtained from a recent work that provides 5hmC-seq data in an AD study conducted by the University of Kentucky Alzheimer’s Disease Research Center with GEO accession number GSE72782 and RNA-seq data with SRA accession number SRA060572 (Bernstein *et al.* 2016). Raw 5hmC-seq data are collected from three prefrontal cortex samples of postmortem AD patients and three controls with similar age, no history of neurological illness, and no significant neuropathology. After data acquisition, read mapping is executed using bowtie2 on the hg19 reference genome, and peak calling is conducted for each sample using MACS2. Peak standardization and read counting for each peak are carried out employing the aforementioned approaches.

For histone modification, our primary focus is on acquiring H3K4me1 and H3K4me3 ChIP-seq data that aligned with the tissue or disease condition associated with the 5hmC-seq data. For “Forebrain Organoid,” we compile aligned bed files of brain-related ChIP-seq data from Roadmap Epigenomics (Kundaje *et al.* 2015) (https://egg2.wustl.edu/roadmap/data/byFileType/alignments/unconsolidated/) (Supplementary Table S1). For “Human Tissues,” we carefully select aligned bed files of ChIP-seq data from Roadmap Epigenomics by ensuring a match between ChIP-seq data and 5hmC-seq data based on tissue type. In cases where tissue-matched ChIP-seq data are unavailable at Roadmap Epigenomics, we retrieve them from the ENCODE portal (https://www.encodeproject.org/). Owing to the absence of matched histone ChIP-seq data for “Hypothalamus” and “Lymph Nodes” in both databases, we exclude the two tissues, resulting in a total of 17 tissues in “Human tissues” for the subsequent analysis (Supplementary Table S2). For “Kentucky AD,” we gather H3K27ac and H3K4me3 ChIP-seq data from the Rush Alzheimer’s Disease Study available on the ENCODE portal (Supplementary Table S3). H3K27ac is used as an alternative for H3K4me1 due to the unavailability of H3K4me1 ChIP-seq data and both H3K27ac and H3K4me1 serve as active enhancer marks. In addition, H3K27ac has been found to correlate with 5hmC (Cui *et al.* 2020). For the AD group, we collect the mapped ChIP-seq bam files from one individual with a matched gender, age, and diagnosed with “Alzheimer’s disease.” For the healthy control group, we obtain the mapped ChIP-seq bam files from one individual with a matched gender, age, and diagnosed with “No Cognitive Impairment.” For chromatin accessibility, we collect tissue-matched DNase-seq data from Roadmap Epigenomics and ENCODE (Supplementary Tables S4 and S5).

### 2.2 Multimodal features

Two types of features are utilized as input for Deep5hmC, which include DNA sequence within the standardized 5hmC peak (i.e. 1 kb) and histone modification in the proximity of the 5hmC peak. The DNA sequence in each 1 kb window undergoes one-hot encoding, adhering to the rule “A”: [1,0,0,0], “C”: [0,1,0,0], “G”: [0,0,1,0] and “T”: [0,0,0,1], which results in a 1000×4 matrix representing the sequence feature. For the histone feature, we extend 10 kb both upstream and downstream of each 5hmC peak and calculate normalized read counts from matched tissue-specific histone ChIP-seq data in a 1 kb window with a sliding size 500 bp, yielding the histone feature with dimensions 1 × 41. In scenarios where n matched ChIP-seq datasets are available, histone features from all datasets are horizontally stacked, resulting in a histone feature with dimensions n × 41.

### 2.3 Creating labeled data for training, validation, and testing

The qualitative prediction is essentially a binary classification task aimed at distinguishing 5hmC peaks from background genomic regions. Specifically, we label standardized 5hmC peaks (i.e. 1 kb) with statistical significance from peak-calling results (False discovery rate (FDR) < 0.05) as positive. To choose peaks in the negative set, we apply a series of selection criteria for genome-wide 1 kb genomic regions of the hg19 reference genome. Initially, negative peaks are required to be within 10 kb distance from the positive ones. Additionally, the density distribution of GC content in the negative peaks must match that of the positive ones. Without loss of generality, we maintain an equal number of positive and negative peaks. As a result, the number of positive peaks ranges from 12 596 to 137 488 with a median of 69 322 among 17 human tissues in “Human Tissue” and from 56 036 to 81 050 with a median of 72 745 for “Forebrain Organoid.” To predict DhMRs in “Kentucky AD,” we start by identifying the 5hmC peaks from all samples in both AD and healthy controls. We merge overlapped peaks, standardize, and calculate the read counts for merged 5hmC peaks. Next, we employ DESeq2 (Love *et al.* 2014) to identify DhMRs. Peaks exhibiting statistical significance are deemed as positive (e.g. FDR < 0.1) and non-significant peaks as considered as negative (e.g. FDR > 0.5). For the quantitative prediction of 5hmC modification, we treat the logarithm of normalized 5hmC reads from both peaks and non-peak genomic regions as the outcome. More details regarding sample size from all datasets can be found in Supplementary Table S6.

### 2.4 Network architecture of Deep5hmC

**Figure 1.**
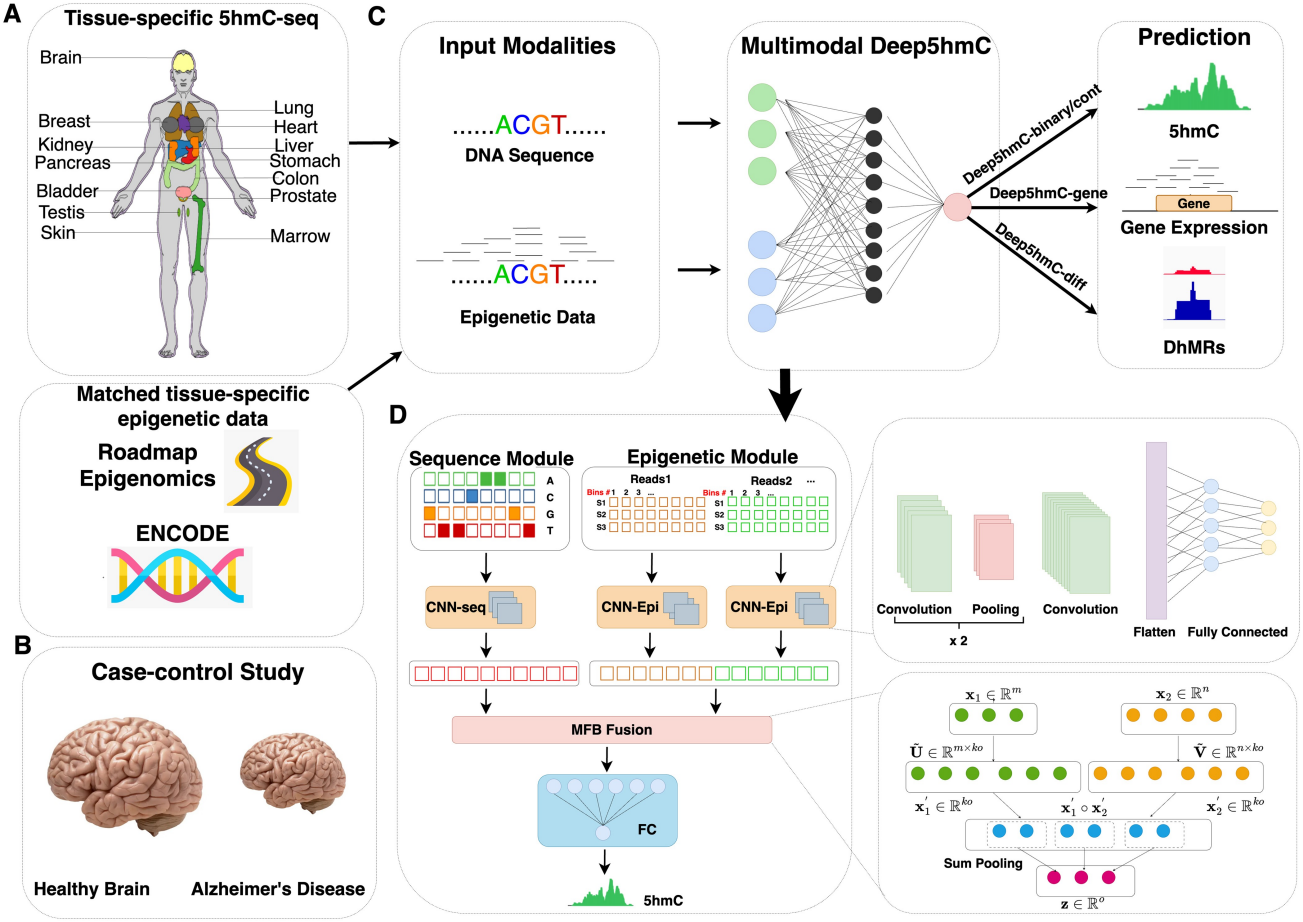
Overview of Deep5hmC. (**A**) The training set of Deep5hmC can be derived from matched 5hmC-seq and other epigenetic data such as histone ChIP-seq or DNase-seq/ATAC-seq from one condition. Specifically, the 5hmC-seq data can be collected from tissue-specific human tissues, which include bladder, brain, breast, heart, kidney, liver, lung, marrow, ovary (female), pancreas, placenta (female), prostate (male), colon (sigmoid), colon (transverse), skin, stomach, and testis (male). The matched tissue-specific epigenetic data, such as histone ChIP-seq data profiling histone modification and DNase-seq/ATAC-seq profiling chromatin accessibility, can be collected from public consortiums such as Roadmap Epigenomics and ENCODE. In this context, Deep5hmC aims to predict genome-wide 5hmC modification in a single condition. (**B**) The training set of Deep5hmC can also be derived from matched 5hmC-seq and ChIP-seq from a case–control study (e.g. Alzheimer’s disease (AD) versus healthy control) for predicting differentially hydroxymethylated regions (DhMRs). (**C**) Deep5hmC is a multimodal deep learning model to improve the prediction of tissue/cell type-specific genome-wide 5hmC modification by leveraging both DNA sequence and epigenetic features such as histone modification and chromatin accessibility. Deep5hmC consists of four modules, including Deep5hmC-binary, Deep5hmC-cont, Deep5hmC-gene, and Deep5hmC-diff. Specifically, Deep5hmC-binary takes the labeled 5hmC peaks and non-peaks as the training set to identify the 5hmC-enriched regions. Deep5hmC-cont takes the normalized read counts in 5hmC peaks and aims to predict the continuous 5hmC modification genome-wide. By leveraging Deep5hmC-cont, Deep5hmC-gene aggregates the predictions of Deep5hmC-cont in the gene bodies as the surrogate for the predicted gene expression. Different from Deep5hmC-binary, Deep5hmC-diff takes the labeled DhMRs/non-DhMRs in a case–control design of 5hmC-seq as the training set to predict genome-wide DhMRs and may discover *de novo* DhMRs. (**D**) Model architecture of Deep5hmC. Deep5hmC consists of both sequence modality and epigenetic modality consisting of their own convolutional neural networks (CNNs) to derive separate feature representations, which will be joined later via the multi-modal factorized bilinear (MFB) pooling fusion layer. The output of the MFB fusion layer will further connect to fully connected layers and the output layer afterward.

Deep5hmC is essentially a multimodal deep learning model, which consists of three crucial components in the network architecture, which includes (i) an encoder module based on two convolutional neural networks (CNNs); (ii) a feature fusion module based on multi-modal factorized bilinear (MFB) pooling approach (Yu *et al.* 2017); and (iii) a prediction module for either binary classification or continuous prediction (Fig. 1D).

The encoder module is composed of two unimodal encoders, each responsible for transforming an individual modality to a high-level feature presentation for further processing by subsequent layers in the model. Specifically, two separate and independent CNNs function as unimodal encoders for DNA sequence and epigenetic feature, respectively. The sequence encoder takes the one-hot encoding DNA sequence as input, consisting of three sequential 1D convolutional layers sharing the same kernel size of 8 and stride of 1, padding of 0, and dilation of 1. The number of filters varies across these layers: 64, 128, and 256. In addition, a max-pooling layer with a kernel size of 4 and a stride of 4 follows each of the first two convolutional layers. The output of the last convolutional layer is flattened and connected to two fully connected layers. On the other hand, the epigenetic encoder takes curated epigenetic features from n epigenetic marks as the input, where each epigenetic mark h is profiled in the dimensions $n_{h}$ × 41. Here, $n_{h}$ represents the number of matched tissues/cell types or biological replicates of the sequencing data. Consequently, the epigenetic encoder takes multimodal epigenetic features as input, with each modality representing a different epigenetic mark (Fig. 1D). Each epigenetic mark has its own CNN to extract the high-level features, comprising three 2D convolutional layers sharing the same kernel size of 3×3, stride of 1, padding of 1, and varying number of filters: 32, 64, and 128. A max-pooling layer with a stride of 2 follows each of the first two convolutional layers. The kernel size for the max-pooling layer depends on the $n_{h}$. For $n_{h}$ equals 1, a 1×2 kernel size is chosen, and otherwise 2×2. Similarly, the output of the last convolutional layer is flattened and is connected to two fully connected layers. Finally, the output from n CNN, corresponding to n epigenetic marks, is concatenated to form the final output of the epigenetic module.

The feature fusion module seamlessly integrates the two feature representations derived from the sequence and epigenetic encoders into a unified representation for subsequent prediction (Fig. 1D). Specifically, we employ MFB (Yu *et al.* 2017), designed to efficiently amalgamate features from diverse modalities. Compared to alternative fusion techniques, MFB excels in capturing intricate interactions among multiple modalities while concurrently reducing computational complexity through factorization. Let $x_{1}\in R^{m}$ denote the feature representation from sequence modality, $x_{2}\in R^{n}$ represent feature representation from epigenetic modality and $z\in R^{o}$ denote the output after fusion module. Notably, o is substantially smaller than both m and n. MFB aims to identify two low-rank factorized matrices $\tilde{U}\in R^{m\times ko}$ and $\tilde{V}\in R^{n\times ko}$, aiming to convert two long vectors of different lengths into two short vectors of the same length ko, where k denotes the latent dimensionality, indicating the degree of factorization. The larger k is, the more original information can be preserved.

(1) $${x'}_{1}=\;{\tilde{U}}^{T}x_{1},\;{x'}_{1}\in R^{ko}$$

(2) $${x'}_{2}=\;{\tilde{V}}^{T}x_{2},\;{x'}_{2}\in R^{ko}$$

The output of MFB fusion can be represented as follows:

(3) $$z=\mathrm{SumPooling}({x'}_{1}\circ {x'}_{2},\;k)$$

where ∘ is the element-wise multiplication of two vectors. Following the SumPooling operation, subsequent layers include power normalization $(\mathrm{sign}\left(z\right){\left|z\right|}^{0.5})\;$and $\ell_{2}\;$normalization $\left(z/∥z∥\right)$ layers. These steps enhance the properties of the fused data, ensuring appropriate scaling and distribution characteristics for subsequent layers.

The prediction module utilizes the output of the MFB fusion layer as the input for the two fully connected layers, which are succeeded by the output layer for the prediction. In the output layer, a single node exists for continuous outcome and two nodes are present for binary outcome. In the case of binary outcome (presence or absence of 5hmc peak), the output undergoes a sigmoid function to yield the prediction probability. For continuous outcome (normalized 5hmC read counts), the output directly serves as the prediction. ReLU serves as the activation function across the entire network, excluding the output layer. Additionally, dropout layers with a rate of 0.5 are strategically incorporated to mitigate overfitting.

### 2.5 Model implementation, training, validation, and testing

Deep5hmC is implemented using PyTorch (Paszke *et al.* 2019) on an NVIDIA A100 GPU system. Utilizing mini-batch gradient descent and the Adam optimizer (Kingma and Ba 2017), the network is optimized for binary outcome using cross-entropy loss and continuous outcome using mean squared error (MSE), respectively. The default learning rate is set to $10^{-3}$. To improve the efficiency of the learning process, warm-up steps and a learning rate decay strategy are incorporated as options. Each model undergoes training for a maximum of 200 epochs, with early stopping implemented if the model performance stagnated over a consecutive 10 epochs. In alignment with the evaluation strategy for DeepSEA, a “cross-chromosomal” strategy is employed to design training, validation, and testing sets. Specifically, 5hmc peaks on chromosomes 8 and 9 constitute the testing set, chromosome 7 serves as the validation set, and the remaining chromosomes form the training set.

## 3 Results

The workflow of the deep learning framework, Deep5hmC is demonstrated in Fig. 1. The labeled training set can be derived from tissue/cell-type specific 5hmC-enriched region (i.e. peaks) in one condition (Fig. 1A) or DhMRs in a case–control study (Fig. 1B) (e.g. disease versus healthy control). Accordingly, the one-hot encoding DNA sequence in peaks or DhMRs serves as the input for the sequence modality (Fig. 1C and D). In addition, epigenetic features such as histone modification and chromatin accessibility are obtained from sequencing data from public consortiums such as ENCODE or Roadmap Epigenomics (Fig. 1A), with matched tissue/cell-type as the 5hmC-seq. Only epigenetic features in the neighborhoods of the 5hmC peaks/DhMRs are considered as the input for the epigenetic modality. The sequence modality and epigenetic modality each go through their own CNNs to derive separate feature representations, which are later joined via the MFB fusion layer. The MFB fusion layer combines features from the two modalities by performing low-rank bilinear pooling, capturing the interactions between modalities efficiently while reducing computational complexity. The output of the MFB fusion layer further connects to fully connected layers and the output layer afterward (more details in Section 2). Depending on the preparation of the training set and prediction mission, the Deep5hmC consists of four modules, including Deep5hmC-binary, Deep5hmC-cont, Deep5hmC-gene, and Deep5hmC-diff. Specifically, Deep5hmC-binary takes the labeled 5hmC peaks and non-peaks as the training set to identify the 5hmC-enriched regions (more details in Section 3.4). Deep5hmC-cont takes normalized read counts in 5hmC peaks and aims to predict the continuous 5hmC modification genome-wide (more details in Section 3.5). By leveraging Deep5hmC-cont, Deep5hmC-gene aggregates the predictions of Deep5hmC-cont in the gene bodies as a surrogate for predicted gene expression (more details in Section 3.6). Different from Deep5hmC-binary, Deep5hmC-diff takes the labeled DhMRs/non-DhMRs in a case–control design of 5hmC-seq as the training set and derives histone features from a similar case–control design of histone ChIP-seq. Deep5hmC-diff aims to predict genome-wide DhMRs and discover *de novo* DhMRs that may be absent in the training set (more details in Section 3.7). Overall, the four modules in the deep learning framework Deep5hmC will provide a comprehensive assessment of genome-wide tissue/cell type-specific 5hmC modification in either a qualitative or quantitative manner as well as genome-wide DhMRs. In addition, it allows the prediction of gene expression using predicted 5hmC modification. The details and evaluation for each module of Deep5hmC will be elaborated in the subsequent sections.

### 3.1 Distribution pattern of histone modification around 5hmC peaks

**Figure 2.**
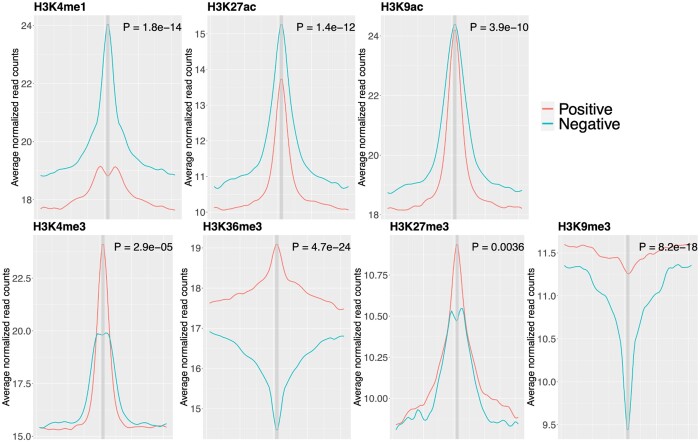
Distribution pattern of histone modification around 5hmC peaks. EB 5hmC peaks are collected from “Forebrain Organoid” 5hmC-seq data and ChIP-seq data in “Brain Angular Gyrus” from seven histone marks are collected from Roadmap Epigenomics. Histone features are obtained and averaged in the neighborhood of all 5hmC peaks for the positive and negative sets, respectively. Specifically, histone features are created by segmenting an extended genomic region of 10 kb both upstream and downstream of each 5hmC peak into 41 1 kb windows with a sliding size of 500 bp and counting reads for each 1 kb windows. For each histone mark, the Kolmogorov–Smirnov test is performed to test the distribution difference of histone features between positive and negative 5hmC peaks and the *P*-value is reported.

We conduct a real data exploration by integrating tissue-matched 5hmC-seq data and histone ChIP-seq data to evaluate the potential of histone modification as informative features for predicting 5hmC modification. Without loss of generality, we gather EB 5hmC peaks from “Forebrain Organoid” and ChIP-seq data in “Brain Angular Gyrus” involving seven histone marks from Roadmap Epigenomics (Supplementary Table S7). The seven histone marks consist of H3K4me1, H3K27ac, and H3K9ac associated with active enhancers; H3K4me3 associated with active promoters; H3K36me3 associated with actively expressed gene bodies; and repressive marks such as H3K9me3 and H3K27me3. To characterize the histone modification patterns around 5hmC modification sites, we acquire and average the histone features with dimensions of 1 × 41 in the neighborhood of each 5hmC peak for the positive and negative sets. The histone features represent essentially normalized 5hmC read counts, which are created by segmenting an extended genomic region of 10 kb both upstream and downstream of each 5hmC peak into 41 1 kb windows, with a sliding size 500 bp and counting reads for each 1 kb windows (more details in Section 2.2). Performing the Kolmogorov–Smirnov test on the two sets of histone features for each histone mark, we find that the distribution of histone features is significantly different between positive and negative 5hmC peaks (*P*-value < 0.05) (Fig. 2). This observation suggests that histone marks are informative for predicting 5hmC modification.

Moreover, the enrichment patterns of the seven histone marks exhibit variations (Fig. 2). Active enhancer marks such as H3K4me1, H3K27ac, and H3K9ac display a consistent pattern, where the histone features are consistently higher in the positive 5hmC peaks than in negative ones. Notably, H3K4me1 shows the most significant differential enrichment of histone features between positive and negative peaks (*P*-value = $1.790\times 10^{-14}$), followed by H3K27ac (*P*-value = $1.380\times 10^{-12}$) and H3K9ac (*P*-value = $3.900\times 10^{-10}$). This observation aligns with the previous findings that 5hmC is significantly enriched in histone marks associated with enhancers, such as H3K4me1 and H3K27ac (Stroud *et al.* 2011). Interestingly, the enrichment pattern is opposite for H3K4me3 compared to the active enhancer marks. H3K4me3 is more enriched in negative 5hmC peaks than in positive ones (*P*-value = $2.923\times 10^{-5}$). A similar trend and shape are observed for H3K27me3 (*P*-value = $3.560\times 10^{-3}$). Although the histone features are higher in negative 5hmC peaks than in positive peaks for both H3K9me3 (*P*-value = $8.235\times 10^{-18})$ and H3K36me3 (*P*-value = $4.708\times 10^{-24})$, the enrichment patterns differ. H3K9me3 exhibits a valley-shaped pattern for both positive and negative peaks, while H3K36me3 shows a peak-shaped pattern for negative peaks and a valley-shaped pattern for positive peaks. Comparing to H3K27me3, which is considered as a temporary repression signal, H3K9me3, another repressive mark viewed as a permanent repression signal, shows the same direction but different enrichment patterns (Kim and Kim 2012). Furthermore, the overall enrichment level of active histone marks is higher than that of repressive histone marks. Taking positive peaks as an example, the average 5hmC read counts in the center of the positive peaks are approximately 24 for H3K4me1, 15 for H3K27ac, 24 for H3K9ac, 20 for H3K4me3 and 14 for H3K36me3 compared to 10 for H3K27me3 and 9 for H3K9me3. Additionally, the change of enrichment from distal windows to the center window of active histone marks is more pronounced than that of repressive histone marks. For positive peaks, the average 5hmC read counts increase by approximately 5 from the most distal 20th window to the center window for H3K4me1, 4 for H3K27ac, 5 for H3K9ac, 5 for H3K4me3, while they decrease by approximately 3 for H3K36me3. In contrast, the average 5hmC read counts increases by only 0.5 for H3K27me3 and decreases by 2 for H3K9me3.

### 3.2 Evaluating the predictive power of Deep5hmC with different histone marks

Motivated by the observation that the enrichment of histone marks shows differential distribution between positive and negative 5hmC peaks, we further explore the predictive power of Deep5hmC using different histone marks in classifying positive and negative 5hmC peaks. Identifying the most influential histone marks is crucial for reducing the model complexity especially when dealing with multiple histone marks in the matched tissues or cell types. Specifically, we choose 5hmC peaks from four developmental stages in “Forebrain Organoid” and ChIP-seq data from all brain regions of seven histone marks in Roadmap Epigenomics (Supplementary Table S1) and evaluate the predictive performance for each histone mark in terms of AUROC and Area Under the Precision-Recall Curve (AUPRC). Consequently, we find that H3K4me1 performs the best, and H3K4me1 and H3K4me3 show consistently higher AUROC than any other individual histone mark across four developmental stages. Moreover, comparing using both H3K4me1 and H3K4me3 to using all seven histone marks, the difference in performance in terms of AUROC is nearly negligible (Supplementary Fig. S1). In addition, the two histone marks are most prevalent across multiple tissue and cell types in consortiums such as ENCODE and Roadmap Epigenomics, while it is difficult to collect all seven histone marks in practical usage. Considering all the above, we only include the two histone marks in Deep5hmC in the subsequent experiments.

### 3.3 Comparing unimodal and multimodal Deep5hmC

**Figure 3.**
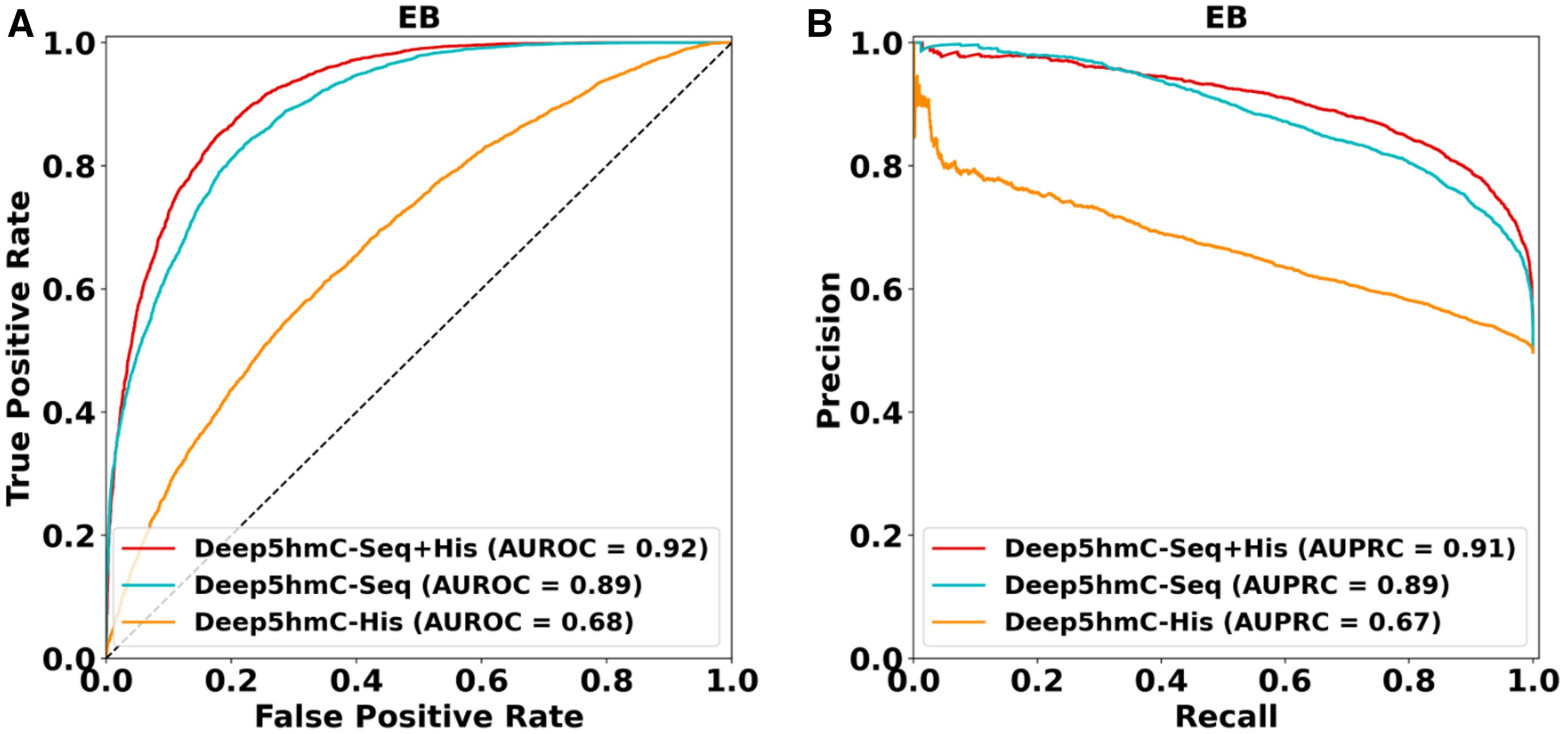
Comparison of unimodal and multimodal Deep5hmC for predicting binary 5hmC modification sites. When using histone modification in the epigenetic modality, two unimodal models of Deep5hmC: Deep5hmC-Seq using only DNA sequence as the model input and Deep5hmC-His using only histone modification as the model input are compared to the default multimodal Deep5hmC-Seq+His using both DNA sequence and histone modification as the model input. 5hmC peaks from the EB stage “Forebrain Organoid” and two histone marks: H3K4me1 and H3K4me3 ChIP-seq data in all brain regions from Roadmap Epigenomics are used as the training set. (**A**) AUROC reported for three compared methods. (**B**) AUPRC reported three compared methods.

As Deep5hmC is a multimodal model comprising both sequence and epigenetic modalities, we conduct an ablation study to demonstrate that incorporating the epigenetic modality leads to improved prediction performance of 5hmC modification. We compare two unimodal models of Deep5hmC: Deep5hmC-Seq and Deep5hmC-His to the default multimodal Deep5hmC-Seq+His. Without loss of generality, we utilize 5hmC peaks from four developmental stages in “Forebrain Organoid” and ChIP-seq data from two histone marks, H3K4me1 and H3K4me3, collected from all brain regions in Roadmap Epigenomics (Supplementary Table S2). At the EB stage, the results indicate that Deep5hmC-Seq+His achieve the best performance with an AUROC of 0.92, followed by Deep5hmC-Seq with an AUROC of 0.89. Deep5hmC-His lags with an AUROC of 0.68 (Fig. 3A). The observation indicates that integrating both sequence and epigenetic modalities indeed improves the prediction performance, although using epigenetic modality alone achieves only moderate predictive power. Similar trends are observed when prediction performance is measured by AUPRC (Fig. 3B). Moreover, consistent results are observed across the other three developmental stages (Supplementary Fig. S2). Therefore, it is evident that multimodal Deep5hmC unequivocally outperforms unimodal Deep5hmC in terms of both AUROC and AUPRC. Given the superior performance of multimodal Deep5hmC-Seq+His, we will use it as the default implementation of Deep5hmC in the subsequent sections.

### 3.4 Evaluating Deep5hmC for predicting binary 5hmC modification sites

To demonstrate the superiority of Deep5hmC to existing methods, we further conduct a comparison between the binary version of Deep5hmC, named Deep5hmC-binary, and competing models, which include DeepSEA-Transfer, DeepSEA-Retrain, and Random Forest to predict the binary 5hmC modification sites (i.e. normalized peaks), using the same training, validation, and testing sets as outlined in the “cross-chromosomal” strategy (more details in Section 2) for both “Forebrain Organoid” and “Human Tissues.” We adopt DeepSEA as the representative of deep learning approaches for its renowned use of genomic sequence to predict epigenetic signals, which has demonstrated superior performance (Zhou and Troyanskaya 2015). Given that DeepSEA is a multi-task model predicting epigenetic signals across various tissues and cell types simultaneously, we customize it into a single-task model in the output layer for a fair comparison to Deep5hmC. In addition, we design two versions of DeepSEA—DeepSEA-Transfer and DeepSEA-Retrain—both sharing the same network architectures as DeepSEA but differing in the training process. Specifically, DeepSEA-Transfer is a transfer learning model built upon the pretrained DeepSEA, with fine-tuning applied exclusively to the last fully connected layer. In contrast, DeepSEA-Retrain starts the model training from the scratch, updating all model parameters. To represent conventional machine learning models, we select Random Forest for its robust performance. Following prior work, we adopt 3-mer frequency of genomic sequence, which results in 64 features for Random Forest (Ahmed *et al.* 2020).

**Figure 4.**
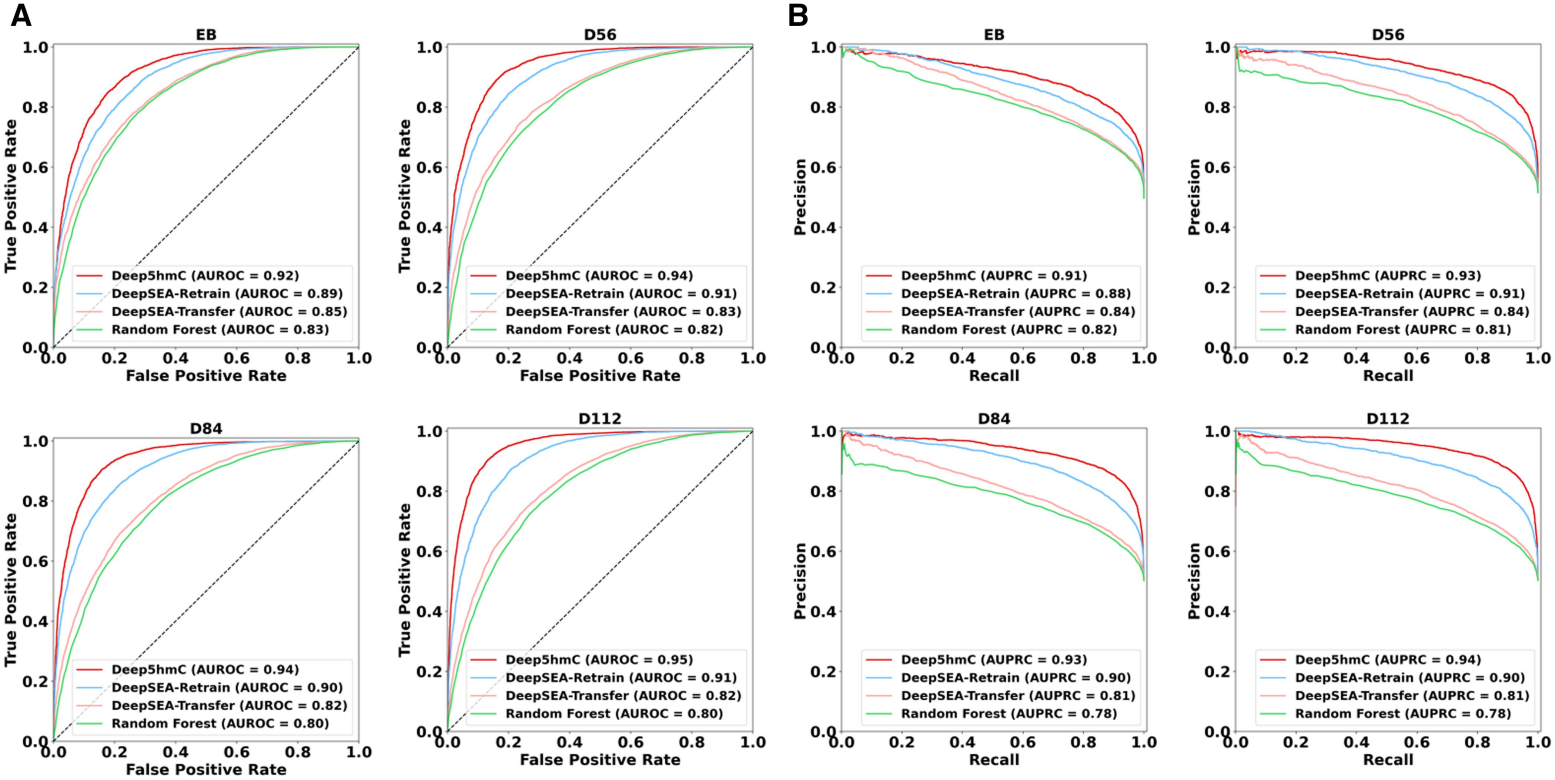
Evaluating Deep5hmC-binary for predicting binary 5hmC modification sites using histone modification in epigenetic modality across four developmental stages in “Forebrain Organoid.” (**A**) AUROC is reported for all compared methods. (**B**) AUPRC is reported for all compared methods.

For “Forebrain Organoid,” we present the AUROC and AUPRC of all models across four developmental stages: day 8 embryoid bodies (EB), day 56 (D56), day 84 (D84), and day 112 (D112) of healthy forebrain organoid (Fig. 4). Consequently, Deep5hmC-binary consistently obtains the highest AUROC (0.92 for EB; 0.94 for D56; 0.94 for D84; 0.95 for D112) among all methods and developmental stages (Fig. 4A). Following closely is DeepSEA-Retrain (0.89 for EB; 0.91 for D56; 0.90 for D84; 0.91 for D112). The observation indicates that enhanced prediction performance can be achieved by leveraging tissue-matched histone modification through a comparison between Deep5hmC-binary and DeepSEA-Retrain, the latter being a CNN model solely relying on genomic sequence as the input. DeepSEA-Retrain outperforms its counterpart DeepSEA-Transfer (AUROC = 0.85 for EB; 0.83 for D56; 0.82 for D84; 0.82 for D112), and Random Forest, which records the lowest overall AUROC (AUROC = 0.83 for EB; 0.82 for D56; 0.80 for D84; 0.80 for D112). The superiority of DeepSEA-Retrain over Random Forest underscores the advantage of the deep learning model in capturing nonlinear and high-order dependencies in the genomic sequence compared to *k*-mer frequency. The observation also suggests that DeepSEA benefits more from retraining the model than relying on the pre-trained model. The initial pretraining of DeepSEA on hundreds of tissue/cell type-specific factors may not be optimal for predicting 5hmC modification, which is another epigenetic factor, emphasizing the importance of context matching, where training and testing data belong to the same domain. Moreover, consistent trends are observed across all methods when evaluated using AUPRC (Fig. 4B).

**Figure 5.**
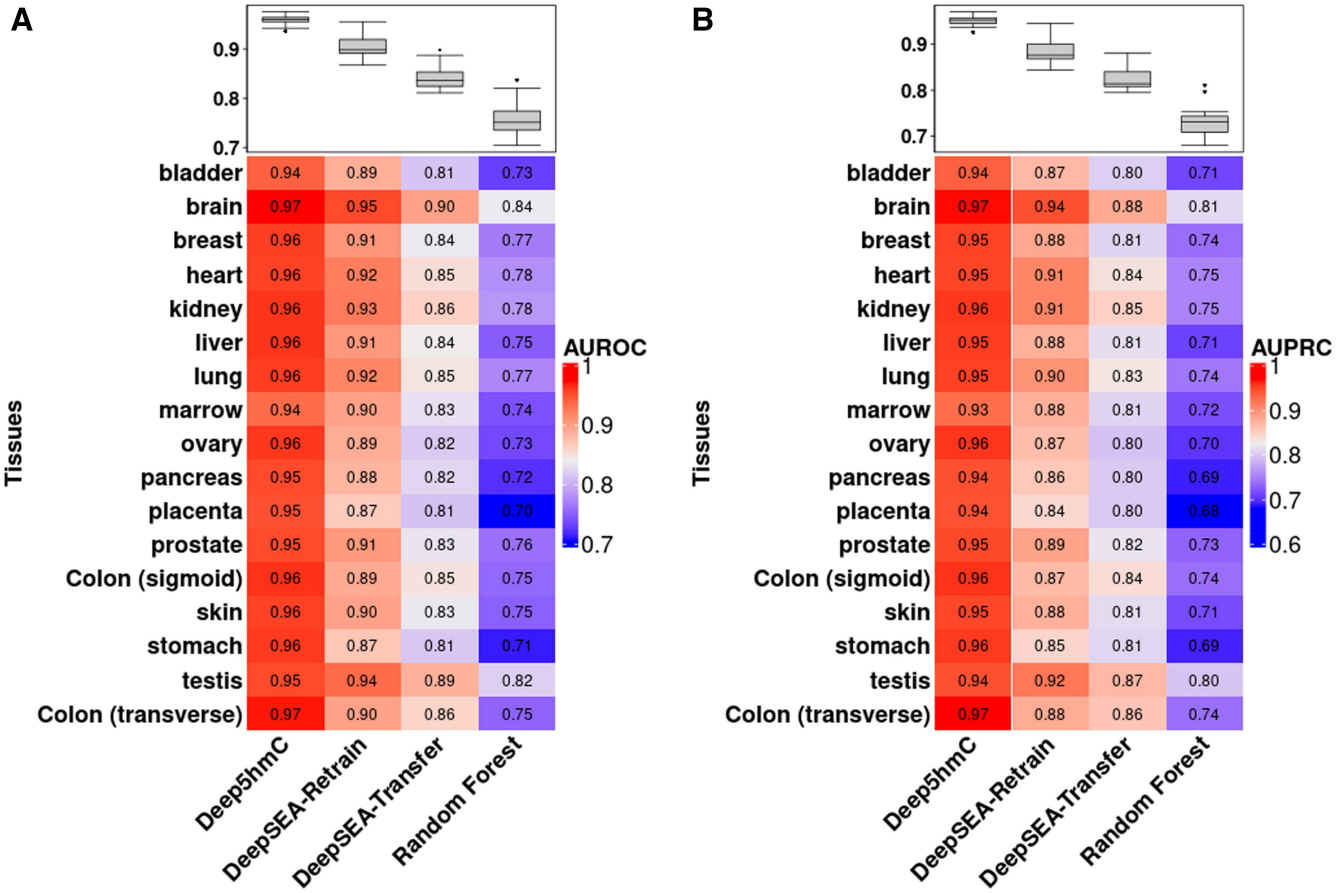
Evaluating Deep5hmC-binary for predicting binary 5hmC modification sites using histone modification in epigenetic modality across 17 human tissues in “Human Tissues.” (**A**) AUROC is reported for all compared methods. (**B**) AUPRC is reported for all compared methods.

For “Human Tissues,” we present both the AUROC and AUPRC of all compared methods across 17 human tissues (Fig. 5). The evaluation of two methods is extended through a Wilcoxon rank-sum test on AUROC/AUPRC across 17 human tissues, aiming to determine the statistical significance of differences in prediction performance. Consequently, Deep5hmC-binary emerges as the top-performed method, followed by DeepSEA-Retrain, DeepSEA-Transfer and Random Forest (median AUROC = 0.96 for Deep5hmC-binary; 0.90 for DeepSEA-Retrain; 0.84 for DeepSEA-Transfer; 0.75 for Random Forest) (Fig. 5A). The superiority of Deep5hmC over other methods is also statistically significant (*P*-value = $1.636\times 10^{-6}$ for Deep5hmC-binary versus DeepSEA-Retrain; $6.972\times 10^{-7}$ for Deep5hmC-binary versus DeepSEA-Transfer; 6.$972\times 10^{-7}$ for Deep5hmC versus Random Forest). Consistent with the findings in “Forebrain Organoid,” DeepSEA-Retrain significantly outperforms DeepSEA-Transfer (*P*-value = $4.190\times 10^{-6})$. Random Forest performs the worst. Moreover, the trend is maintained when assessing prediction performance using AUPRC (Fig. 5B). Conclusively, the comprehensive evaluation focusing on tissue-specific predictions underscores that Deep5hmC-binary possesses a distinct advantage over existing methods concerning binary 5hmC modification sites.

### 3.5 Evaluating Deep5hmC for predicting continuous 5hmC modification

Using the same two datasets as aforementioned in predicting binary 5hmC modification sites, we extend the evaluation to the continuous version of Deep5hmC, named Deep5hmC-cont, along with other competing methods for predicting continuous 5hmC modification, quantified by normalized 5hmC read counts. To mitigate the impact of outliers and normalize the count data toward a normal distribution, we perform a log transformation on the 5hmC read counts. Evaluation of prediction performance is conducted using Spearman correlation coefficient (*R*) and MSE, which are calculated between the observed 5hmC read counts and predicted ones on the logarithm scale.

**Figure 6.**
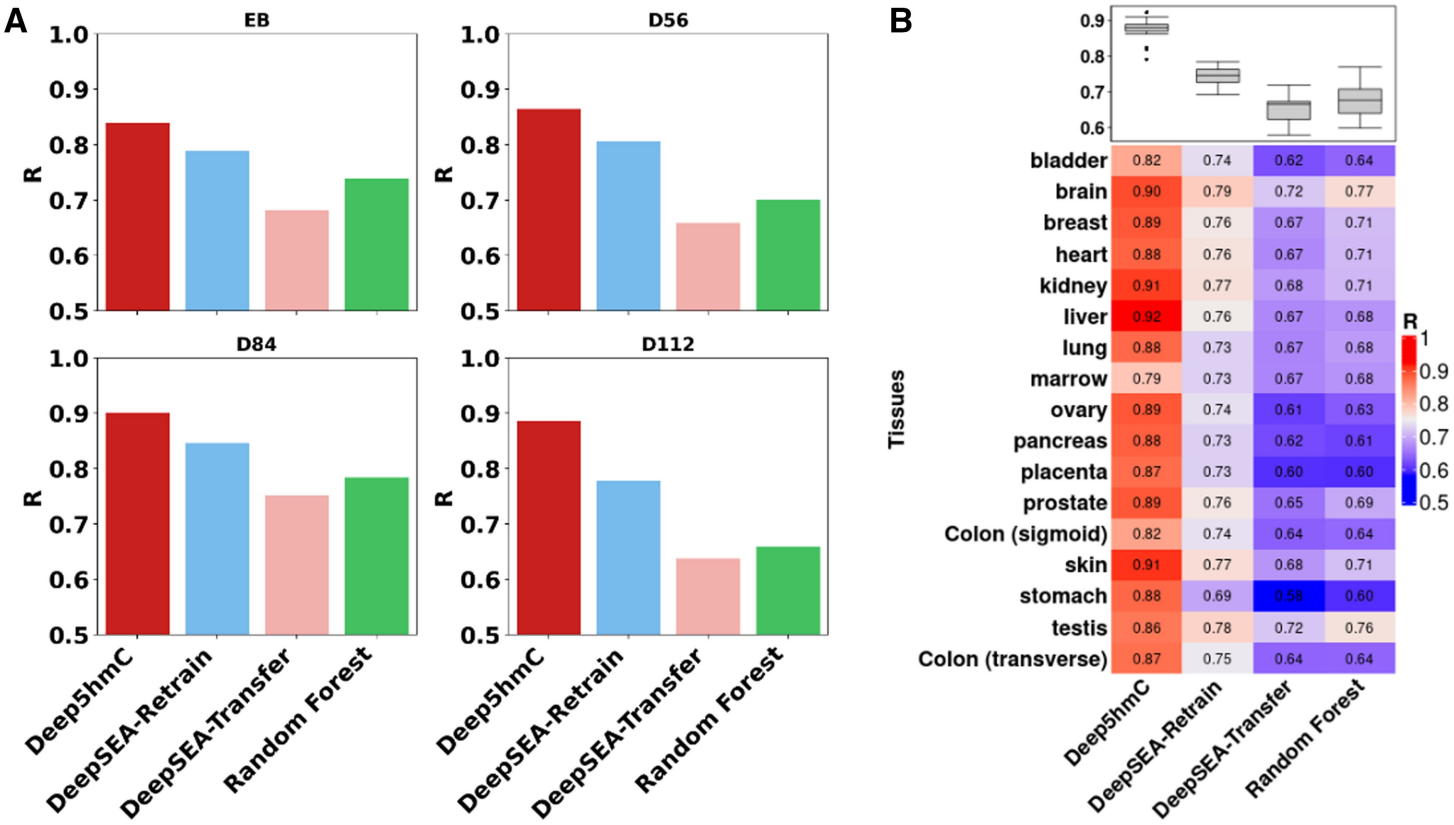
Evaluating Deep5hmC-cont for predicting continuous 5hmC modification using histone modification in epigenetic modality. (**A**) Spearman correlation coefficient (*R*) is reported for all compared methods across four developmental stages in “Forebrain Organoid.” (**B**) *R*s are reported for all compared methods across 17 human tissues in “Human Tissues”.

For “Forebrain Organoid,” Deep5hmC-cont demonstrates the highest *R* across all developmental stages (*R* = 0.839 for EB; 0.864 for D56; 0.901 for D84; 0.886 for D112) (Fig. 6A). Following closely is DeepSEA-Retrain, showing better performance than Random Forest (*R*s = 0.789, 0.806, 0.846, and 0.778 for DeepSEA-Retrain; 0.739, 0.700, 0.784 and 0.659 for Random Forest). Notably, DeepSEA-Transfer remains potent, yet it exhibits the least favorable performance with *R*s of 0.682, 0.658, 0.752, and 0.638 across four developmental stages. In addition, Deep5hmC-cont obtains the smallest MSE in three out of four developmental stages (Supplementary Fig. S3A).

For “Human Tissues,” Deep5hmC-cont excels by emerging as the top-performed method in all tissues in terms of *R*s and achieving the highest median of *R* across 17 tissues (median *R* = 0.881 for Deep5hmC-cont; 0.746 for DeepSEA-Retrain; 0.665 for DeepSEA-Transfer; *R* = 0.677 for Random Forest) (Fig. 6B). DeepSEA-Retrain ranks second, while Random Forest and DeepSEA-Transfer have comparable performance. Upon comparing the Deep5hmC-cont to other methods using the Wilcoxon rank-sum test on the *R*s from 17 tissues, we find that Deep5hmC-cont enjoys a substantial advantage over DeepSEA-Retrain, DeepSEA-Transfer and Random Forest (*P*-value = $7.013\times 10^{-7}$ for Deep5hmC-cont versus DeepSEA-Retrain; $7.027\times 10^{-7}$ for Deep5hmC-cont versus DeepSEA-Transfer; $6.999\times 10^{-7}$for Deep5hmC-cont versus Random Forest). Once again, DeepSEA-Retrain significantly performs better than DeepSEA-Transfer (*P*-value = $9.976\times 10^{-7})$. Additionally, Deep5hmC-cont achieves the smallest median of MSE, and smallest MSE in 14 out of the 17 tissues, as well as the second smallest MSE in 3 out of the 17 tissues (Supplementary Fig. S3B). These observations affirm that Deep5hmC-cont accurately predicts the tissue-specific continuous 5hmc modification, showcasing its improvement over other methods.

### 3.6 Evaluating Deep5hmC for predicting gene expression

The positive correlation between 5hmC modification in the gene body and gene expression has been demonstrated in both mouse brain and human tissues (Mellén *et al.* 2012, He *et al.* 2021). To quantify the predictive power for gene expression using 5hmC modification, we introduce Deep5hmC-gene, a module within the Deep5hmC framework, designed to predict gene expression by leveraging continuous predicted 5hmC modification from Deep5hmC-cont. Specifically, Deep5hmC-gene employs a three-step approach. First, it segments each gene body into nonadjacent 1 kb windows. For gene bodies less than 1 kb or with the last window less than 1 kb, padding is applied to ensure each window is 1 kb. Next, Deep5hmC-gene utilizes the pre-trained Deep5hmC-cont to predict the 5hmC counts for each 1 kb window. Finally, it aggregates all predicted 5hmC counts within each gene body to generate the predicted gene expression. To evaluate the effectiveness of Deep5hmC-gene in predicting gene expression, we evaluate it on both “Forebrain Organoid” and “Human Tissues,” which provide a comprehensive set of tissue-specific paired 5hmC-seq data and RNA-seq data. Gene expression measured by RNA-seq data, in terms of read counts, serves as the gold standard. The evaluation is based on the Spearman correlation coefficient (*R*) calculated between predicted and observed gene expression. In addition, we report *R*s calculated between predicted and observed 5hmC read counts in all gene bodies, as the predicted gene expression is quantified by predicted 5hmC read counts in gene bodies.

**Figure 7.**
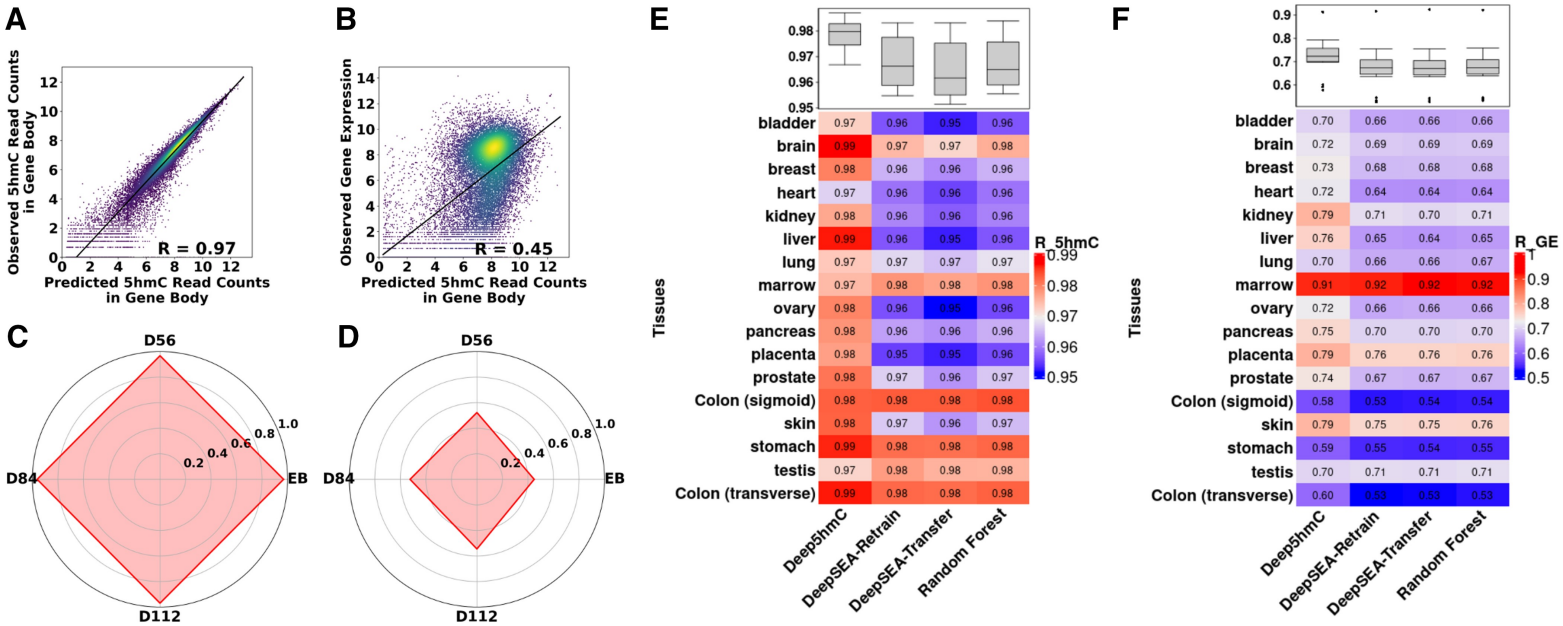
Evaluating Deep5hmC-gene for predicting gene expression. (**A**) A correlation plot between the predicted and observed 5hmC read counts in all gene bodies for EB stage in “Forebrain Organoid.” (**B**) A correlation plot between predicted and observed gene expression for EB stage in “Forebrain Organoid.” (**C**) Spearman correlation coefficient (*R*) calculated between the predicted and observed 5hmC read counts in all gene bodies for four developmental stages in “Forebrain Organoid.” (**D**) *R*s calculated between the predicted and observed gene expression for four developmental stages in “Forebrain Organoid.” (**E**) *R*s calculated between the predicted and observed 5hmC read counts in all gene bodies for 17 human tissues in “Human Tissues.” (**F**) *R*s calculated between the predicted and observed gene expression for 17 human tissues in “Human Tissues”.

As a result, Deep5hmC-gene achieves a high *R*-value of 0.970 between the predicted and observed 5hmC read counts in all gene bodies for EB in “Forebrain Organoid” (Fig. 7A). Leveraging this accurate prediction of 5hmC in gene bodies, Deep5hmC-gene obtains a substantial *R*-value of 0.449 between predicted and observed gene expression for EB in “Forebrain Organoid” (Fig. 7B). Extending the analysis to all four developmental stages in the “Forebrain Organoid” reveals consistent results, with Deep5hmC-gene accurately predicting 5hmC read counts in gene bodies (*R* within the range of 0.962–0.970) (Fig. 7C) and gene expression (*R* within the range of 0.449–0.545) (Fig. 7D). The MSE calculated between predicted and observed 5hmC read counts in gene bodies, as well as between predicted and observed gene expression for “Forebrain Organoid” can be found in the Supplementary Fig. S4A and B.

Moreover, we benchmark Deep5hmC-gene against other competing methods for “Human Tissues.” All approaches demonstrate high prediction accuracy for 5hmC read counts in gene bodies in terms of *R* (Fig. 7E). Deep5hmC-gene exhibits comparable performance to other methods, with a median *R*-value of 0.980 compared to 0.966 for DeepSEA-Retrain, 0.962 for DeepSEA-Transfer and 0.965 for Random Forest. Notably, Deep5hmC-gene exhibits the smallest median of MSE (Supplementary Fig. S4C). The prediction accuracy for gene expression of all methods declines and shows tissue-specific variability (Fig. 7F). Specifically, Deep5hmC-gene performs best, achieving the highest median of *R*-value of 0.723 compared to 0.674 for DeepSEA-Retrain, 0.671 for DeepSEA-Transfer, and 0.674 for Random Forest. Notably, the *R*-values of most tissues are above 0.60 for all methods, indicating that using 5hmC read counts can accurately predict gene expression. Once again, Deep5hmC-gene achieves the smallest median of MSE between the predicted and observed gene expression for “Human Tissues” (Supplementary Fig. S4D). Overall, the exploration demonstrates that leveraging 5hmC read counts in gene bodies facilitates accurate prediction of gene expression, potentially linking DNA methylation and gene expression in a tissue-specific gene regulatory context.

### 3.7 Evaluating Deep5hmC for predicting differential hydroxymethylated regions

Thus far, we have demonstrated the efficacy of Deep5hmC in predicting tissue-specific 5hmC modification, quantified by both 5hmC peak and continuous 5hmC reads. However, extending our study to a case–control design allows us to explore differential hydroxymethylated regions (DhMRs). In this scenario, DhMRs are regions enriched or present only in one disease or treatment condition, while absent or depleted in the control condition, or vice versa. Identified DhMRs can be potential biomarkers for disease prevention, diagnosis, and treatment. We formularize the DhMR identification as a binary classification task, where each genomic region (i.e. 1 kb) is labeled and predicted as DhMR or non-DhMR. This module for predicting DhMRs is named “Deep5hmC-diff.” Deep5hmC-diff utilizes Deep5hmC-His modality, masking Deep5hmC-Seq modality, as the genomic sequence is shared between two conditions in the same genomic regions. The training set for Deep5hmC-diff is created by performing differential peak analysis using tools such as DESeq2 (Love *et al.* 2014) and ChIPComp (Chen *et al.* 2015) on merged 5hmC peaks from all samples in both case and control conditions. Subsequently, Deep5hmC-diff is trained and tested using the labeled DhMRs.

To demonstrate the feasibility of Deep5hmC-diff, we focus on an AD study, specifically “Kentucky AD,” which profiles 5hmC-seq for 3 AD and 3 healthy controls. We employ DESeq2 (Love *et al.* 2014) to identify DhMRs (e.g. FDR < 0.1) and non-DhMRs (e.g. FDR > 0.5), resulting in 4330 differential DhMRs and 4025 non-DhMRs. The histone features are derived from H3K27ac and H3K4me3 ChIP-seq data from Rush Alzheimer’s Disease Study, available on the ENCODE portal (Sloan *et al.* 2016). H3K27ac is used as an alternative to H3K4me1 given the unavailability of H3K4me1 data, and both H3K27ac and H3K4me1 are active enhancer marks. For each histone mark, we select one ChIP-seq data with matched gender, age for the 5hmC-seq in the AD group, and diagnosed with “Alzheimer’s disease” and another ChIP-seq data with matched gender, age for the 5hmC-seq in the healthy control group, and diagnosed with “No Cognitive Impairment” (Supplementary Table S3).

**Figure 8.**
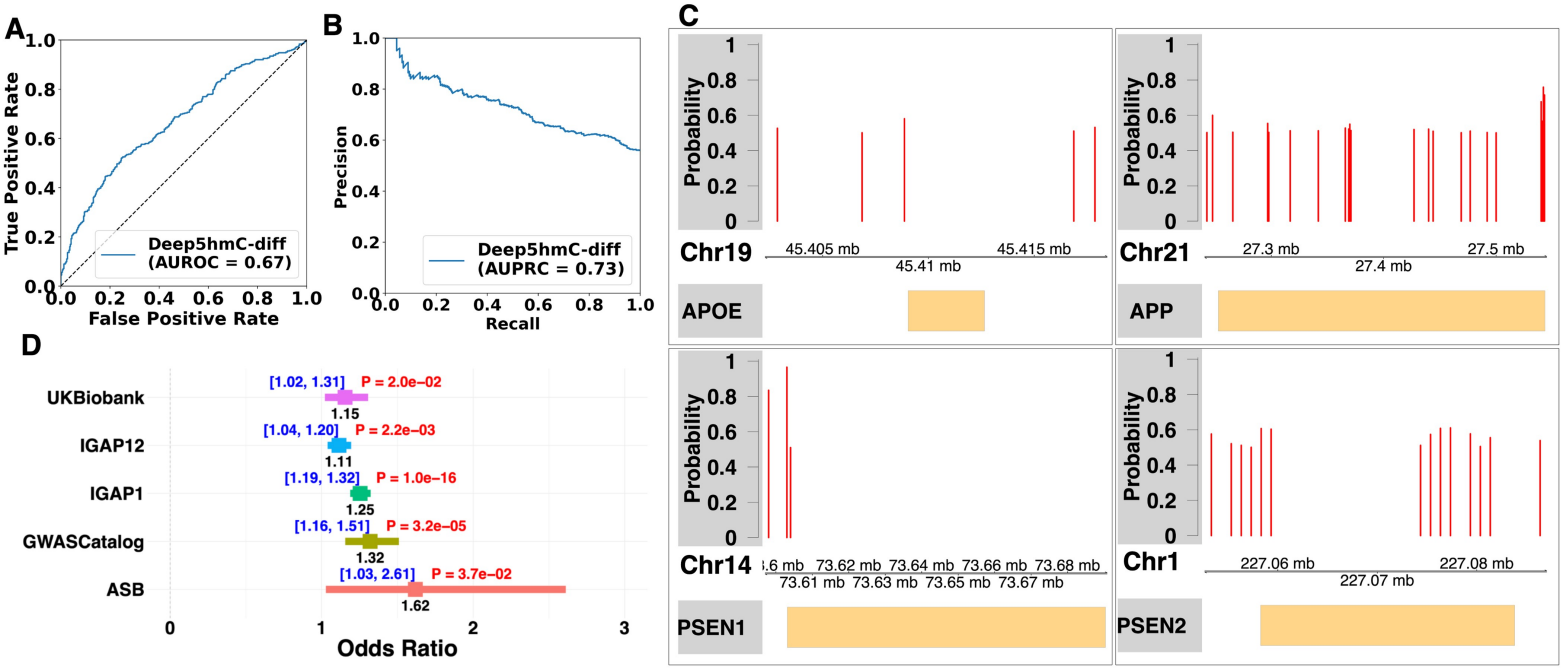
Applying Deep5hmC-diff in a case–control study of Alzheimer’s disease (AD). (**A**) AUROC is reported for predicting differential hydroxymethylated regions (DhMRs) between AD patients and healthy controls in “Kentucky AD.” (**B**) AUPRC is reported for predicting DhMRs between AD patients and healthy controls. (**C**) Distribution of identified *de novo* DhMRs in three causal genes associated with early onset AD, which include *PSEN1* (chr14:73603143–73690399), *PSEN2* (chr1:227058273–227083804), and *APP* (chr21:27252861–27543138) as well as one causal gene *APOE* (chr19:45409039–45412650) associated with late onset AD. (**D**) SNP enrichment analysis to evaluate the enrichment of AD-associated SNPs in *de novo* DhMRs. Positive SNPs are collected from five sources including UK Biobank. Association Results Browser (ARB), GWASCatalog, International Genomics of Alzheimer’s Project (IGAP) stage1 and combined stage1 & 2.

We employ the same “cross-chromosomal” strategy to create training, validation, and testing sets. Deep5hmC-diff achieves an AUROC of 0.67 (Fig. 8A) and AUPRC of 0.73 (Fig. 8B), demonstrating predictive power for identifying DhMRs by leveraging histone modification data. There is potential for further improvement by incorporating additional histone marks or other epigenetic factors such as chromatin accessibility and transcription factor binding. In addition to evaluating Deep5hmC-diff using the “cross-chromosomal” strategy, we extend its application by conducting a genome-wide screening for *de novo* DhMRs, which may not be present in the 5hmC-seq data potentially due to lacking sufficient sequencing depths or technical bias, etc. For this purpose, we utilize all labeled DhMRs and non-DhMRs peaks from “Kentucky AD” to train the Deep5hmC-diff model. The entire human genome is then segmented into non-overlapping 1 kb windows, serving as the testing set. Each 1 kb window, considered a candidate genomic region, is assigned a predictive probability of being a DhMR or not, using a cutoff at 0.5. The distributions of *de novo* DhMRs are found to be consistent with those from “Kentucky AD” across different genomic features, including Introns, Intergenic Regions, Promoters, Exons, immediate Downstream, 5UTRs and 3UTRs (Supplementary Fig. S5A and B). Of particular interest is the evaluation of whether Deep5hmC-diff can identify *de novo* DhMRs within key functional genomic sites associated with AD. We focus on three causal genes associated with early onset AD, which include *PSEN1* (chr14:73603143–73690399), *PSEN2* (chr1:227058273–227083804), and *APP* (chr21:27252861–27543138) as well as one causal gene *APOE* (chr19:45409039–45412650) associated with late onset AD. The predicted probability within the gene bodies and upstream and downstream 5 kb of the gene bodies are plotted (Fig. 8C). Deep5hmC-diff successfully identifies multiple *de novo* DhMRs with more DhMR found for *APP* and *PSEN2* than *APOE* and *PSEN1*. As 5hmC modification is positively correlated with gene expression, we conduct differential expression analysis to validate the identified differential *de novo* DhMRs. We collect matched RNA-seq data for AD and healthy controls from “Kentucky AD” and perform differential expression analysis using DESeq2 (Love *et al.* 2014). Consequently, three out of the four causal genes show differential expression (FDR = 0.019 for *APP*; 0.031 for *PSEN1*; 0.028 for *PSEN2*), supporting the findings of predicted DhMRs in the three genes. These observations indicate that Deep5hmC-diff can be a valuable tool for identifying novel DhMRs in a case–control study.

Single nucleotide polymorphism (SNP) enrichment analysis is designed to assess the enrichment of diseases/traits-associated GWAS SNPs or eQTLs within tissue/cell type-specific epigenetic regions. The analysis is crucial for identifying disease/trait-associated cell types, providing functional annotation, and elucidating the role of GWAS SNPs or eQTLs (Kundaje *et al.* 2015, Chen *et al.* 2016, Chen and Qin 2017, Chen *et al.* 2019, Wang and Chen 2022, Agarwal *et al.* 2023). Recent AD studies have extensively employed SNP enrichment analysis to assess the enrichment of AD-associated SNPs in DhMRs, which helps unravel the functional implications of these AD-associated SNPs in AD pathogenesis (Bernstein *et al.* 2016). Building on these insights, we conduct SNP enrichment analysis to evaluate the enrichment of AD-associated SNPs in *de novo* DhMRs, which are defined as genome-wide 1 kb candidate regions with a predictive probability greater than 0.5 contrasting with non-DhMRs.

Statistically significant AD-associated SNPs, considered positive SNPs, are gathered from five resources, containing summary statistics from GWAS conducted in AD. The first set of positive SNPs is derived from a study named “genome-wide association study by proxy (GWAX),” comprising 1302 significant SNPs from UK Biobank (*P*-value < 1 × $10^{-4}$) (Liu *et al.* 2017). The second set is obtained from GWASCatalog (https://www.ebi.ac.uk/gwas/), including 1108 significant SNPs (*P*-value < 1 × $10^{-4}$). The third set is sourced from the Association Results Browser (ARB) (https://www.ncbi.nlm.nih.gov/projects/gapplus/sgap_plus.htm), containing 111 significant SNPs (*P*-value < 1 × $10^{-4}$). The other two sets of positive SNPs are acquired from the International Genomics of Alzheimer’s Project (IGAP) stage1 and combined stage1 & 2, harboring 6225 and 3687 significant SNPs, respectively (*P*-value < 1 × $10^{-4}$) (Lambert *et al.* 2013). Moreover, only SNPs in the noncoding regions are considered given the majority of GWAS SNPs are located within noncoding regions. To establish a reliable control group, we generate negative control SNPs at a ratio of 10:1 compared to the positive set, following the strategy from previous work (Chen *et al.* 2016, Chen and Qin 2017). Subsequently, for each variant set, we tally the number of positive/negative SNPs within DhMRs/non-DhMRs and construct a 2 by 2 contingency table. Fisher’s exact test is then employed to calculate the odds ratio (OR), confidence interval (CI), and *P*-value for the table (Fig. 8D). The results reveal that all five sets of positive SNPs exhibit enrichment in the *de novo* DhMRs (OR > 1 and *P*-value < 0.05), suggesting a crucial role of AD-associated SNPs in the pathogenesis of AD through their enrichment in DhMRs.

### 3.8 Evaluating Deep5hmC for predicting 5hmC modification using chromatin accessibility

The improved prediction performance of Deep5hmC is attributed to its integration of epigenetic modality, which includes histone modification in the above sections. Besides histone modification, other epigenetic factors are of interest to be explored for their predictive potential. Cell type-specific 5hmC profiles have been found associated with transcriptional abundance and chromatin accessibility across human hematopoiesis (Nakauchi *et al.* 2022). We hereby collect tissue-matched DNase-seq, which profiles chromatin accessibility, from both Roadmap Epigenomics and ENCODE to evaluate the prediction performance of multimodal Deep5hmC. We start with comparing unimodal and multi-modal Deep5hmC using chromatin accessibility in the epigenetic modality across four developmental stages in “Forebrain Organoid.” We find that Deep5hmC-Seq+CA consistently leads the performance in terms of both AUROC and AUPRC compared to Deep5hmC-Seq and Deep5hmC-CA, which indicates leveraging chromatin accessibility in the epigenetic modality also helps improve the prediction performance (Supplementary Fig. S6). Next, we benchmark this version of multi-modal Deep5hmC, Deep5hmC-Seq+CA, abbreviated as Deep5hmC, against DeepSEA-Retrain, DeepSEA-Transfer and Random Forest on four developmental stages in “Forebrain Organoid.” For predicting binary 5hmC modification sites, Deep5hmC consistently outperforms other methods by achieving the highest AUROC and AUPRC (Supplementary Fig. S7). For predicting continuous 5hmC modification, Deep5hmC obtains the highest Spearman correlation coefficient (*R*) across the four developmental stages, and smallest MSE at D84 and D112 and comparable MSE with DeepSEA-Retrain at D56 (Supplementary Fig. S8). While benchmarking all methods on 17 human tissues in “Human Tissues,” we find that Deep5hmC is superior to other methods by obtaining the largest median of AUROC and AUPRC for binary prediction; and largest median of *R* and smallest median of MSE for continuous prediction (Supplementary Fig. S9). Notably, Deep5hmC is consistently the top-performed method across all 17 human tissues for binary prediction in terms of both AUROC and AUPRC; and for continuous prediction in terms of *R*s. In addition, Deep5hmC achieves the smallest MSE in 9 out of the 17 tissues, as well as the second smallest MSE in 6 out of the 17 tissues for continuous prediction. The above results demonstrate that leveraging chromatin accessibility in the epigenetic modality can also empower Deep5hmC, indicating the robustness and versatility of Deep5hmC to accommodate different types of epigenetic features. In practice usage of Deep5hmC, users have the flexibility to choose histone modification and chromatin accessibility depending on the data availability.

## 4 Conclusion and discussion

In this study, we present a comprehensive deep-learning framework named Deep5hmC, designed to predict the genome-wide landscape of 5hmC. Deep5hmC comprises four distinct modules tailored to specific prediction tasks: Deep5hmC-binary for predicting binary 5hmC peaks; Deep5hmC-cont for predicting continuous 5hmC modification; Deep5hmC-gene for predicting gene expression; Deep5hmC-diff for predicting differential hydroxymethylated regions (DhMRs). Notably, Deep5hmC stands out as a multi-modal deep learning model, which incorporates both DNA genomic sequence and other epigenetic features such as histone modification and chromatin accessibility to improve the prediction for genome-wide qualitative and quantitative 5hmC modification. The decision to include histone modality stems from a thorough exploration of real data, involving tissue-matched histone ChIP-seq data from seven histone marks in a specific one brain region and 5hmC-seq data profiled in EB from forebrain organoid. This exploration reveals distinct distribution patterns between 5hmC peaks and non-peak genomic regions in histone modifications of both active and repressive histone marks, suggesting the informative nature of histone modification in predicting 5hmC modification. Notably, H3K4me1 and H3K4me3 are identified as the most informative histone marks. To accommodate the epigenetic modality, Deep5hmC employs n CNNs, each corresponding to a different histone mark. The output from the epigenetic modality is then integrated with the output from the sequence modality through the MFB fusion layer, resulting in a joint embedding for subsequent predictions. Using an illustrative example with tissue-matched 5hmC-seq data at four developmental stages from forebrain organoid and H3K4me1 and H3K4me3 ChIP-seq collected from Roadmap Epigenomics, we demonstrate that the multi-modal Deep5hmC outperforms both single-modal counterparts utilizing only DNA sequence or relying solely on histone modification.

We further employ the multi-modal version of Deep5hmC as the default model for comparative analysis against existing methods, which include Random Forest and two variants of DeepSEA involving fine-tuning or retraining on two comprehensive datasets. These datasets encompass a broad collection of 5hmC-seq across human tissues. One dataset, named “Forebrain Organoid,” comprises matched 5hmC-seq and RNA-seq from four stages during fetal brain development. The other dataset, named “Human Tissues,” includes matched 5hmC-seq and RNA-seq from 17 diverse human tissues. Through an evaluation using the “cross-chromosomal” strategy, Deep5hmC-binary emerges as superior to existing methods, achieving the highest AUROC and AUPRC for predicting binary 5hmC modification sites. Similarly, Deep5hmC-cont attains the highest Spearman correlation coefficient and smallest MSE for predicting continuous 5hmC modification. Moreover, leveraging the predictions from pre-trained Deep5hmC-cont, Deep5hmC-gene aggregates all predicted 5hmC counts within the gene body, accurately predicting the gene expression for both “Forebrain Organoid” and “Human Tissues.” This observation underscores the regulatory connection between DNA hydroxymethylation and gene expression in a tissue-specific context. We also explore the chromatin accessibility, another epigenetic feature reported to be associated with 5hmC profile. Consequently, we find that the inclusion of chromatin accessibility alone in the epigenetic modality can improve the prediction performance of multi-modal Deep5hmC compared to its single-modal counterparts. In addition, the multi-modal Deep5hmC version that incorporates chromatin accessibility also outperforms competing methods for predicting both binary and continuous 5hmC modification.

In addition to predicting 5hmC modification in a single healthy tissue, Deep5hmC-diff enables the prediction of differential hydroxymethylated regions (DhMRs) in a case–control design, where the regions, enriched or present only in one disease/treatment condition but depleted or absent in the control condition (or vice versa), are of particular interest. To demonstrate the feasibility, Deep5hmC-diff is applied to the “Kentucky AD” study with matched 5hmC-seq and RNA-seq data for both AD patients and healthy controls. The results not only showcase the accurate prediction of DhMRs using the “cross-chromosomal” strategy but also successfully identify genome-wide *de novo* DhMRs. Notably, multiple *de novo* DhMRs are found in AD causal genes such as *APP*, *APOE*, *PSEN1*, and *PSEN2*. These findings are further supported by differential expression analysis using the matched RNA-seq data. In addition, significant SNPs reported to be associated with AD from various studies are found to be enriched in DhMRs, indicating a potential role of DhMRs in AD pathogenesis. Overall, these discoveries underscore the potency and broad applications of Deep5hmC in 5hmC-seq analysis.

Several promising extensions of current work are envisioned. First, the 5hmC-seq data used to train Deep5hmC lack single-base resolution. The incorporation of high-resolution 5hmC data from advanced technologies, such as Tet-assisted bisulfite sequencing (TAB-Seq) and Oxidative bisulfite sequencing (oxBS-Seq), provides an opportunity to extend Deep5hmC’s capability to predict 5hmC modification at the single-base level. To achieve this, we intend to adapt and develop large language models, accommodating the significantly increased training sample size and addressing spatial correlation among single-base modification sites. In this work, we select the histone marks based on their prediction performance in a heuristic way. We plan to develop an explainable version of Deep5hmC utilizing attention mechanisms in the future work, which may not only automatically select the informative histone marks but also enable the identification of functional interactions between 5hmC and other epigenetic marks, shedding light on their interplay in the regulation of gene expression. This approach seeks to provide a more interpretable and nuanced understanding of the complex relationships within the epigenetic landscape. Third, while we have considered histone modification and chromatin accessibility in the epigenetic modality, other epigenetic features can be further explored in the multi-modal deep learning framework such as transcription factor binding (Murga-Garrido *et al.* 2021). This expansion aims to enhance prediction performance by considering a more comprehensive set of epigenetic features. Lastly, for the continuous prediction for both 5hmC modification and RNA-seq count data, we apply log transformation for the count data, which may not be optimal. In future work, we will redesign the deep learning framework by modeling the count data using Poisson or negative binomial distribution, and predict the continuous outcome on the count scale directly (Czado *et al.* 2009).

## Supplementary Material

btae528_Supplementary_Data

## Data Availability

Deep5hmC is available via https://github.com/lichen-lab/Deep5hmC.
