# Supplementary material for: Deep5hmC: predicting genome-wide 5-hydroxymethylcytosine landscape via a multimodal deep learning model
Source: Bioinformatics. 2024 Aug 28;40(9):btae528. doi: 10.1093/bioinformatics/btae528 (PMC11379467; doi:10.1093/bioinformatics/btae528)
Supplement: btae528_Supplementary_Data [file btae528_supplementary_data.pdf]

Supplementary Materials for Deep5hmC: Predicting genome-wide 5-Hydroxymethylcytosine landscape via multimodal deep learning model

Supplementary Figures

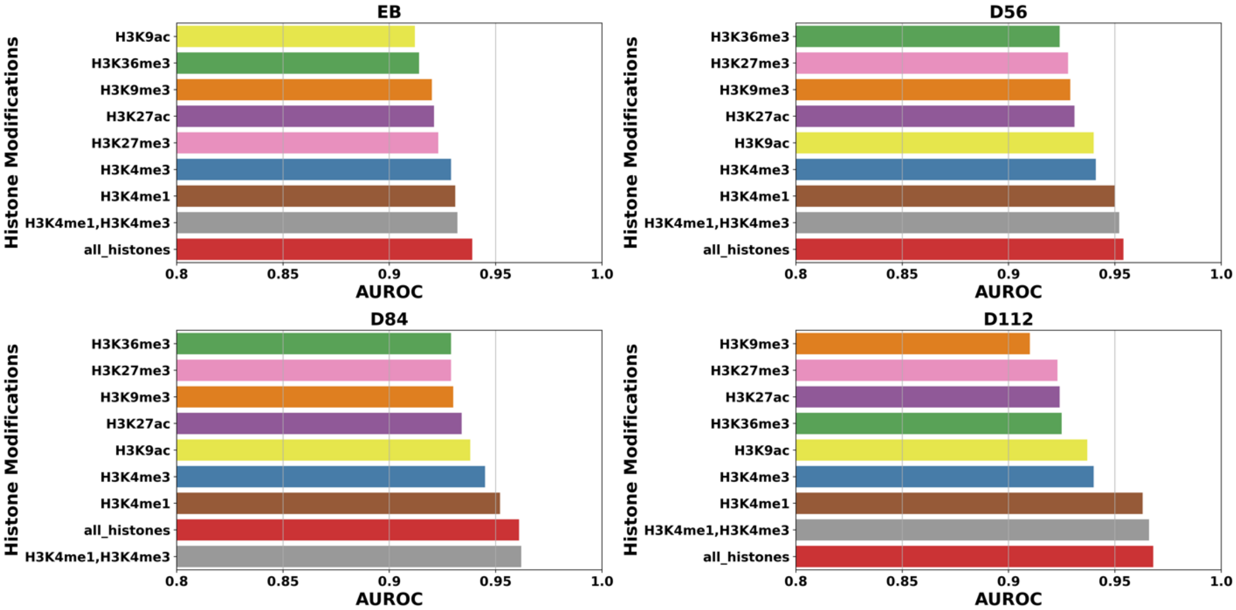

Supplementary Figure S1. Evaluating predictive performance of Deep5hmC with different histone marks across four developmental stages in "Forebrain organoid".

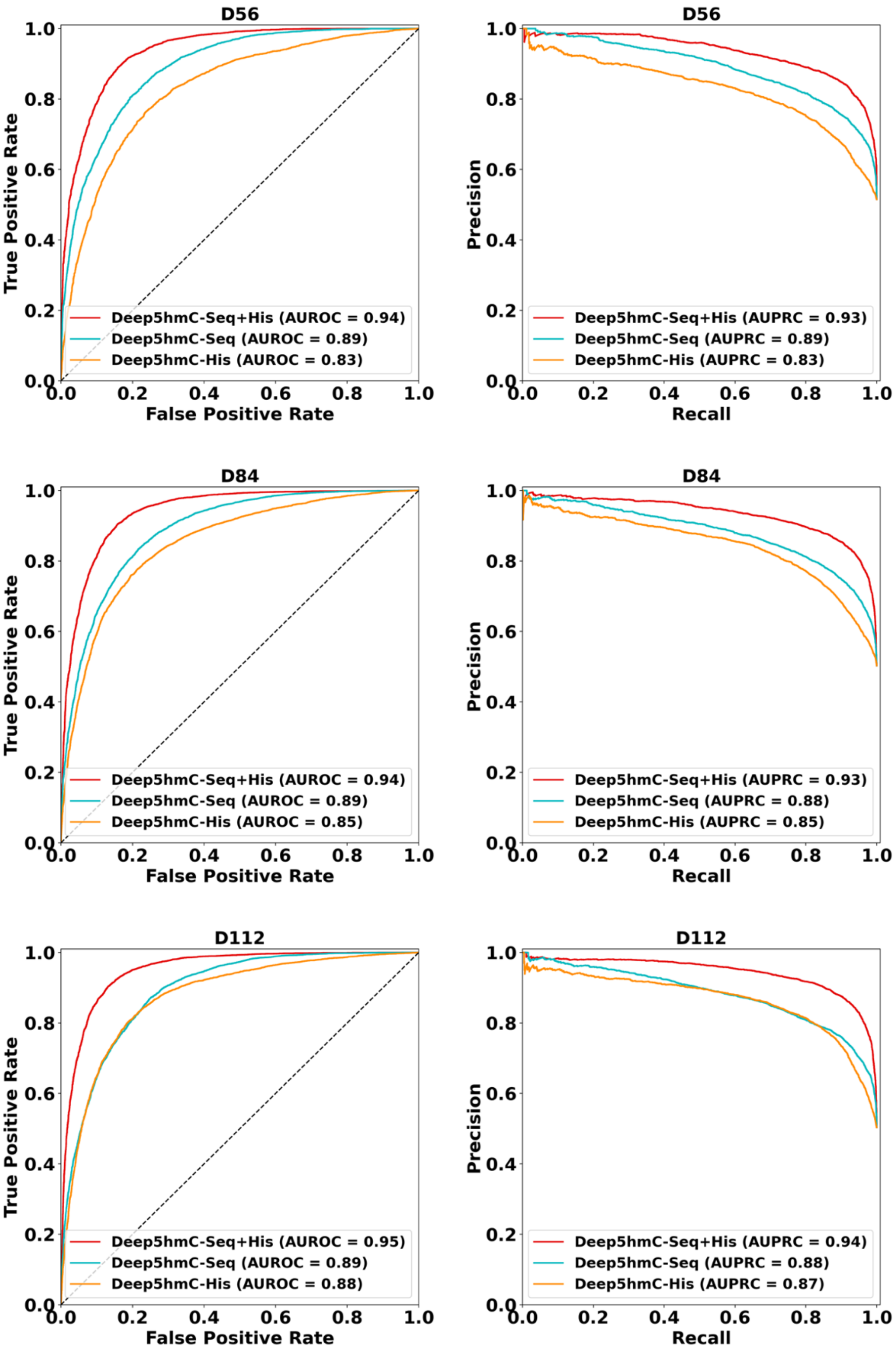

Supplementary Figure S2. Comparison of unimodal and multimodal Deep5hmC for predicting binary 5hmC modification sites. When using histone modification in the epigenetic modality, two unimodal models of Deep5hmC: Deep5hmC-Seq using only DNA sequence as the model input and Deep5hmC-His using only histone modification as the model input are compared to the default multimodal

Deep5hmC-Seq+His using both DNA sequence and histone modification as the model input. 5hmC peaks from the D56, D84, D112 developmental stages at "Forebrain Organoid" and two histone marks: H3K4me1 and H3K4me3 ChIP-seq data in all brain regions from Roadmap Epigenomics are used as the training set. **A.** AUROC reported for three compared methods. **B.** AUPRC reported for three compared methods.

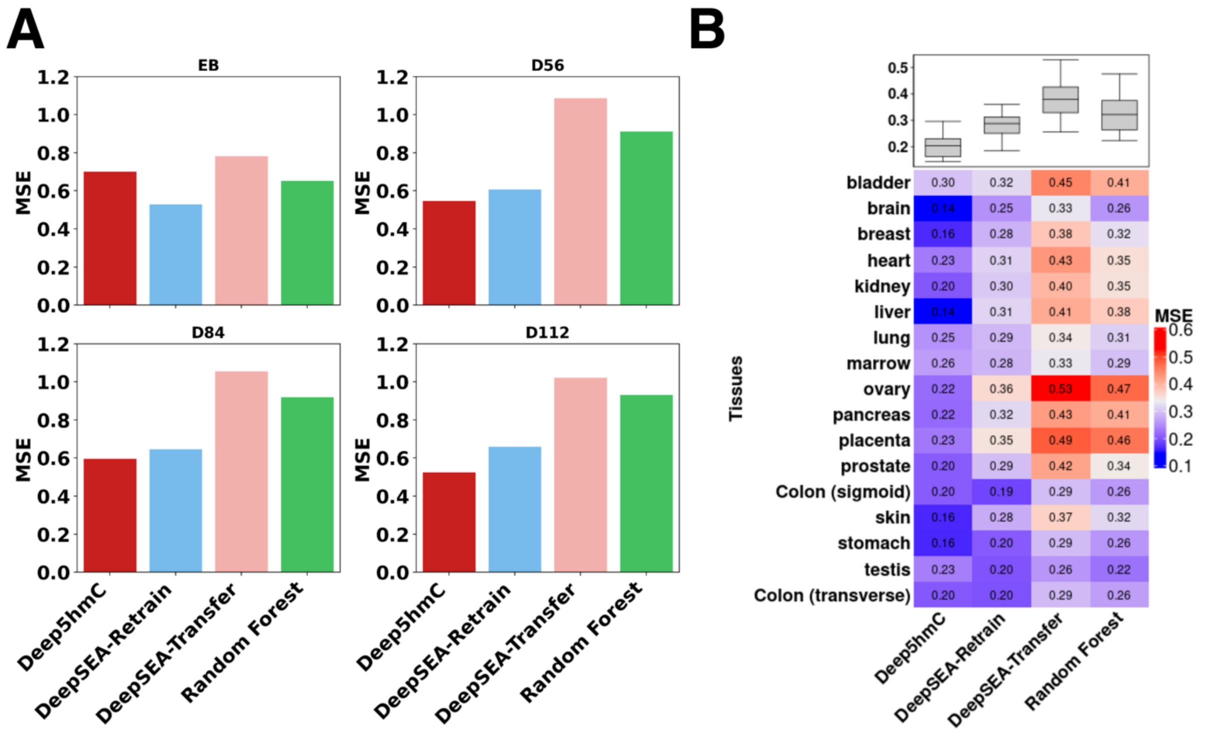

Supplementary Figure S3. Evaluating Deep5hmC-cont for predicting continuous 5hmC modification using histone modification in epigenetic modality. A. Mean squared error (MSE) is reported for all compared methods across four developmental stages in "Forebrain Organoid". **B.** MSE is reported for all compared methods across 17 human tissues in "Human Tissues".

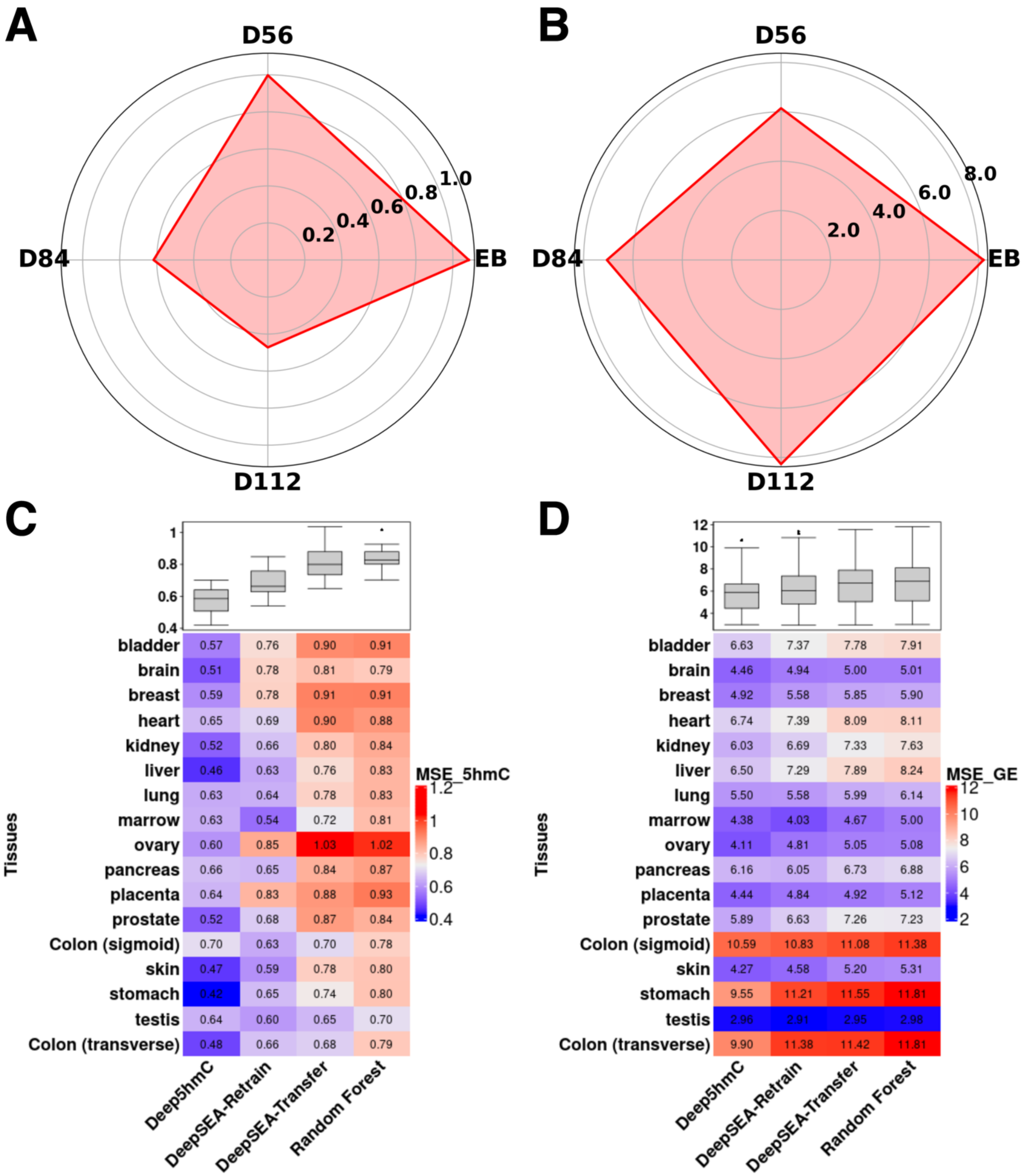

Supplementary Figure S4. Evaluating Deep5hmC-gene for predicting gene expression. A. Mean squared error (MSE) is calculated between the predicted and observed 5hmC read counts in all gene bodies for four developmental stages in "Forebrain Organoid". **B.** MSE is calculated between the predicted and observed gene expression for four developmental stages in "Forebrain Organoid". **C.** MSE is calculated between the predicted and observed 5hmC read counts in all gene bodies for 17 human tissues in "Human Tissues". **D.** MSE is calculated between the predicted and observed gene expression for 17 human tissues in "Human Tissues".

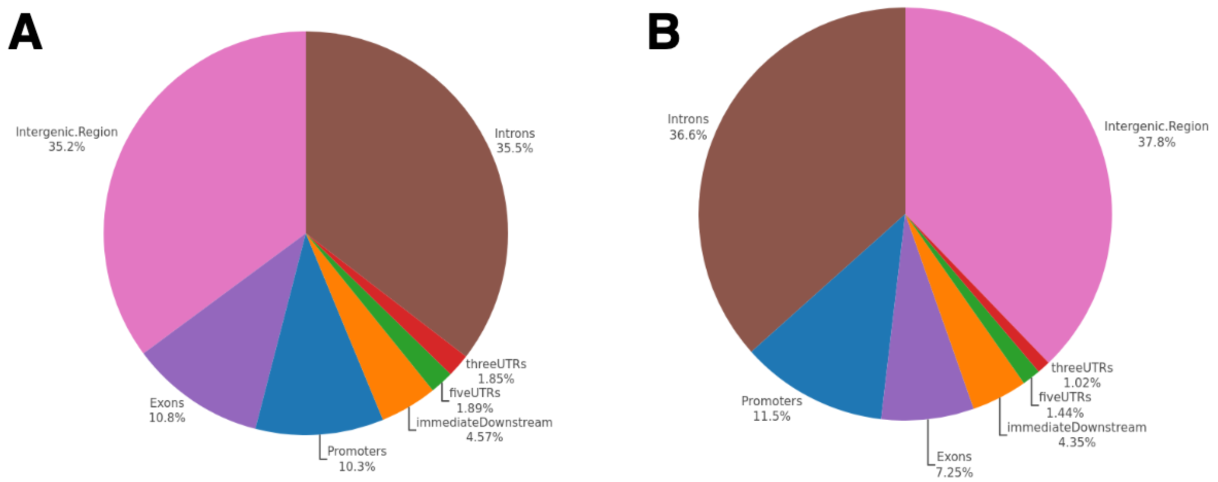

Supplementary Figure S5. Comparing the distribution of DhMRs in the training set of "Kentucky AD" to genome-wide *de novo* DhMRs across different genomic features. A. The distribution of DhMRs in the training set of "Kentucky AD" across different genomic features. **B.** The distribution of genome-wide *de novo* DhMRs across different genomic features.

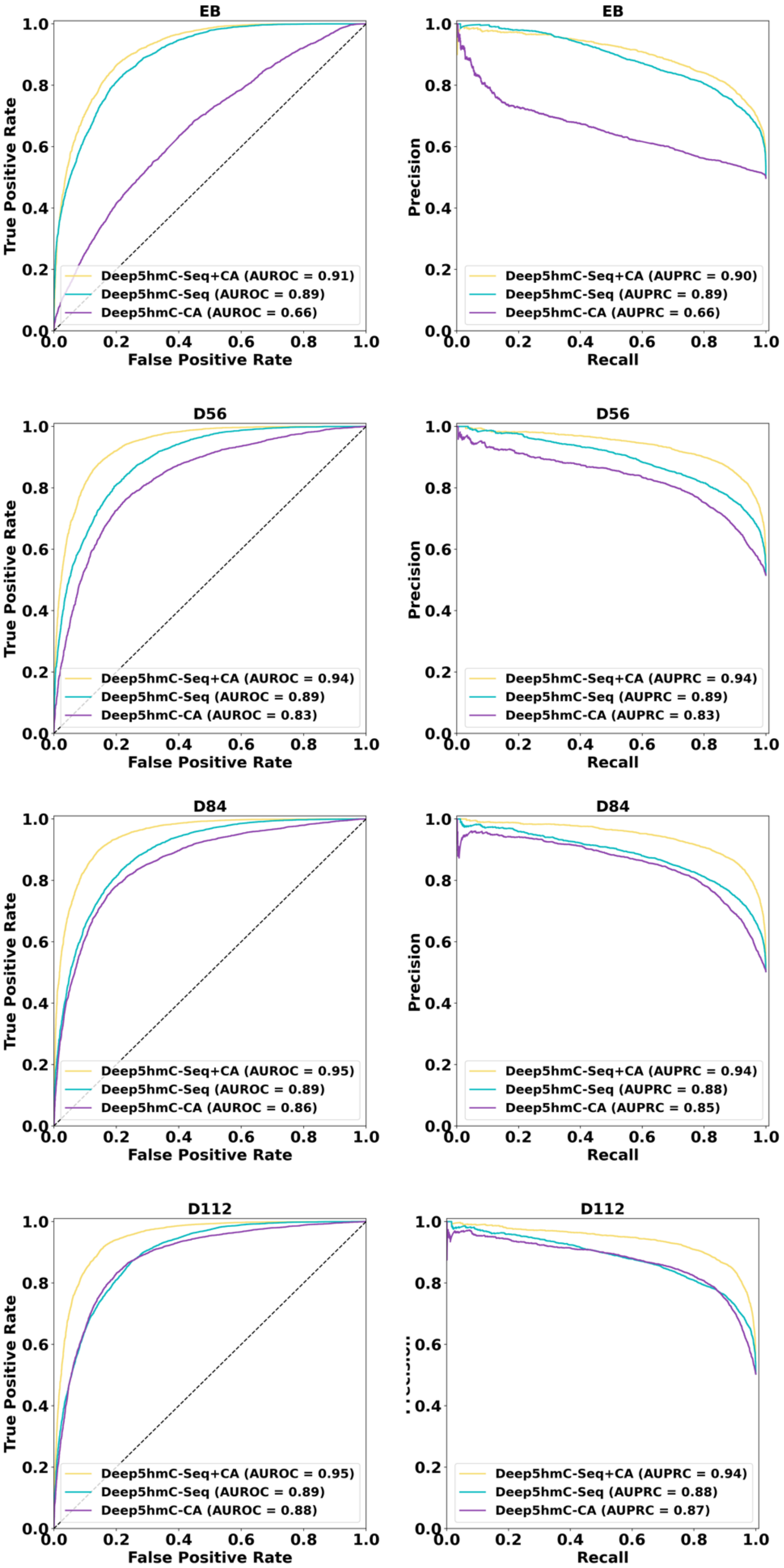
EB
True Positive Rate
False Positive Rate
Deep5hmC-Seq+CA (AUROC = 0.91)
Deep5hmC-Seq (AUROC = 0.89)
Deep5hmC-CA (AUROC = 0.66)
EB
Precision
Recall
Deep5hmC-Seq+CA (AUPRC = 0.90)
Deep5hmC-Seq (AUPRC = 0.89)
Deep5hmC-CA (AUPRC = 0.66)
D56
True Positive Rate
False Positive Rate
Deep5hmC-Seq+CA (AUROC = 0.94)
Deep5hmC-Seq (AUROC = 0.89)
Deep5hmC-CA (AUROC = 0.83)
D56
Precision
Recall
Deep5hmC-Seq+CA (AUPRC = 0.94)
Deep5hmC-Seq (AUPRC = 0.89)
Deep5hmC-CA (AUPRC = 0.83)
D84
True Positive Rate
False Positive Rate
Deep5hmC-Seq+CA (AUROC = 0.95)
Deep5hmC-Seq (AUROC = 0.89)
Deep5hmC-CA (AUROC = 0.86)
D84
Precision
Recall
Deep5hmC-Seq+CA (AUPRC = 0.94)
Deep5hmC-Seq (AUPRC = 0.88)
Deep5hmC-CA (AUPRC = 0.85)
D112
True Positive Rate
False Positive Rate
Deep5hmC-Seq+CA (AUROC = 0.95)
Deep5hmC-Seq (AUROC = 0.89)
Deep5hmC-CA (AUROC = 0.88)
D112
Recall
Deep5hmC-Seq+CA (AUPRC = 0.94)
Deep5hmC-Seq (AUPRC = 0.88)
Deep5hmC-CA (AUPRC = 0.87)

Supplementary Figure S6. Comparison of unimodal and multimodal Deep5hmC using chromatin accessibility. When using chromatin accessibility in the epigenetic modality, two unimodal models of Deep5hmC: Deep5hmC-Seq using only DNA sequence as the model input and Deep5hmC-CA using only chromatin accessibility as the model input are compared to the default multimodal Deep5hmC-Seq+CA using both DNA sequence and chromatin accessibility features as the model input. 5hmC peaks from "Forebrain Organoid" and DNase-seq data in all brain regions from Roadmap Epigenomics are used as the training set. **A.** AUROC reported for three compared methods. **B.** AUPRC reported for three compared methods.

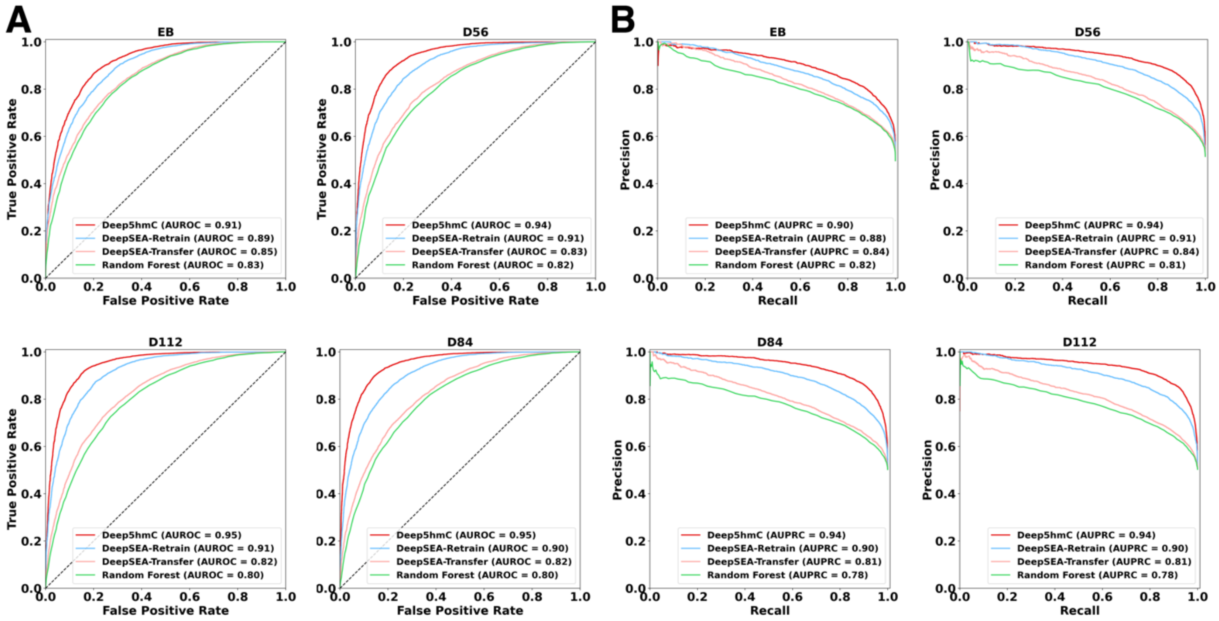

Supplementary Figure S7. Evaluating Deep5hmC using chromatin accessibility for predicting binary 5hmC modification sites across four developmental stages in "Forebrain Organoid". A. AUROC is reported for all compared methods. **B.** AUPRC is reported for all compared methods.

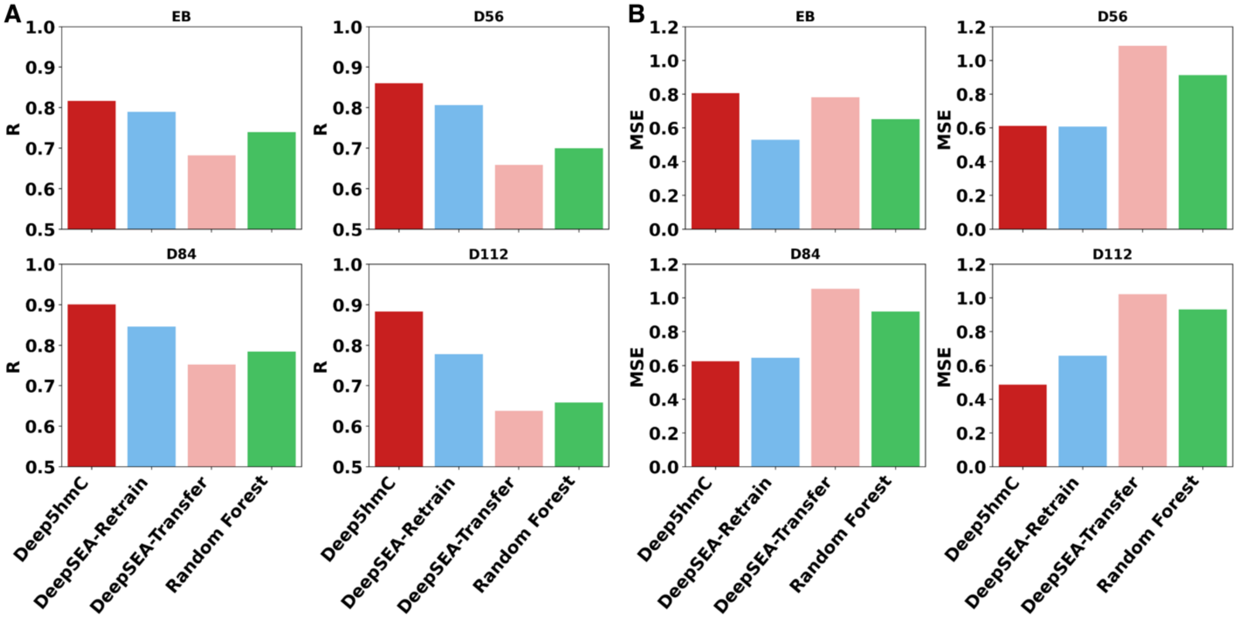

Supplementary Figure S8. Evaluating Deep5hmC using chromatin accessibility for predicting continuous 5hmC modification across four developmental stages in "Forebrain Organoid". A. Spearman Correlation Coefficient (R) is reported between the predicted and observed 5hmC modification for all compared methods for all compared methods. **B.** Mean squared error (MSE) is calculated between the predicted and observed 5hmC modification for all compared methods.

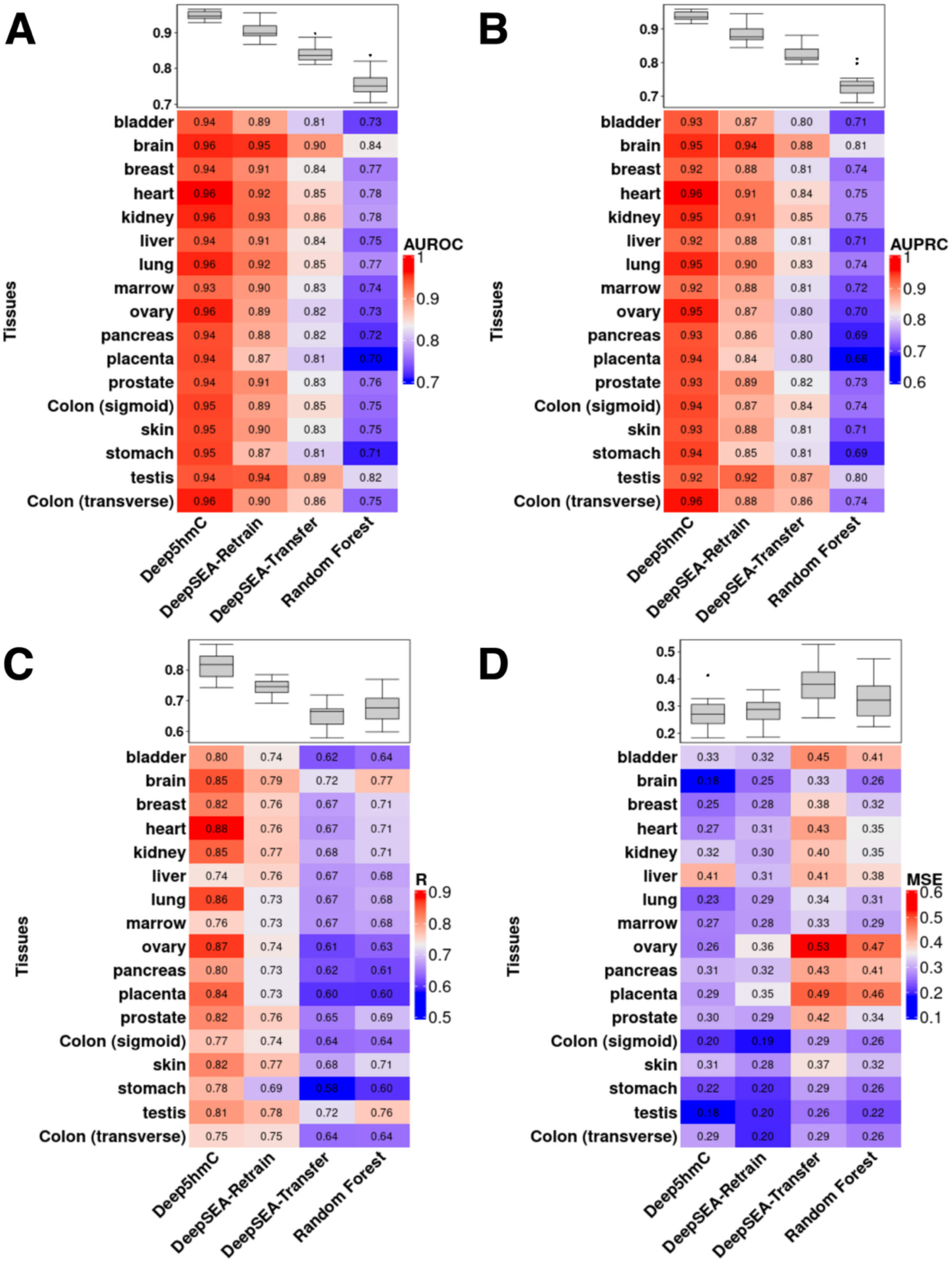

Supplementary Figure S9. Evaluating Deep5hmC using chromatin accessibility for predicting binary/continuous 5hmC modification across 17 human tissues in "Human Tissues". A. AUROC is reported for all compared methods. **B.** AUPRC is reported for all compared methods**. C.** Spearman correlation coefficient (R) is reported between the predicted and observed 5hmC modification for all compared methods. **D.** MSE is calculated between the predicted and observed 5hmC modification for all compared methods.

Supplementary Tables

Histone Mark	ChIP-seq Data Source
H3K4me1	BI.Brain_Angular_Gyrus.H3K4me1.112.filt.tagAlign.gz;<br>BI.Brain_Angular_Gyrus.H3K4me1.149.filt.tagAlign.gz;<br>BI.Brain_Anterior_Caudate.H3K4me1.112.filt.tagAlign.gz;<br>BI.Brain_Anterior_Caudate.H3K4me1.149.filt.tagAlign.gz;<br>BI.Brain_Cingulate_Gyrus.H3K4me1.112.filt.tagAlign.gz;<br>BI.Brain_Cingulate_Gyrus.H3K4me1.149.filt.tagAlign.gz;<br>BI.Brain_Hippocampus_Middle.H3K4me1.112.filt.tagAlign.gz;<br>BI.Brain_Hippocampus_Middle.H3K4me1.149.filt.tagAlign.gz;<br>BI.Brain_Hippocampus_Middle.H3K4me1.150.filt.tagAlign.gz;<br>BI.Brain_Inferior_Temporal_Lobe.H3K4me1.112.filt.tagAlign.gz;<br>BI.Brain_Inferior_Temporal_Lobe.H3K4me1.149.filt.tagAlign.gz;<br>BI.Brain_Mid_Frontal_Lobe.H3K4me1.112.filt.tagAlign.gz;<br>BI.Brain_Mid_Frontal_Lobe.H3K4me1.149.filt.tagAlign.gz;<br>BI.Brain_Substantia_Nigra.H3K4me1.112.filt.tagAlign.gz;<br>BI.Brain_Substantia_Nigra.H3K4me1.149.filt.tagAlign.gz;<br>BI.Fetal_Brain.H3K4me1.UW_H22676.filt.tagAlign.gz;<br>UCSF-UBC.Brain_Germinal_Matrix.H3K4me1.HuFGM01.filt.tagAlign.gz;<br>UCSF-UBC.Brain_Germinal_Matrix.H3K4me1.HuFGM02.filt.tagAlign.gz;<br>UCSF-UBC.Fetal_Brain.H3K4me1.HuFNSC01.filt.tagAlign.gz;<br>UCSF-UBC.Fetal_Brain.H3K4me1.HuFNSC02.filt.tagAlign.gz
H3K27ac	BI.Brain_Angular_Gyrus.H3K27ac.112.filt.tagAlign.gz;<br>BI.Brain_Angular_Gyrus.H3K27ac.149.filt.tagAlign.gz;<br>BI.Brain_Anterior_Caudate.H3K27ac.112.filt.tagAlign.gz;<br>BI.Brain_Anterior_Caudate.H3K27ac.149.filt.tagAlign.gz;<br>BI.Brain_Cingulate_Gyrus.H3K27ac.112.filt.tagAlign.gz;<br>BI.Brain_Cingulate_Gyrus.H3K27ac.149.filt.tagAlign.gz;<br>BI.Brain_Hippocampus_Middle.H3K27ac.112.filt.tagAlign.gz;<br>BI.Brain_Hippocampus_Middle.H3K27ac.149.filt.tagAlign.gz;<br>BI.Brain_Hippocampus_Middle.H3K27ac.150.filt.tagAlign.gz;<br>BI.Brain_Inferior_Temporal_Lobe.H3K27ac.112.filt.tagAlign.gz;<br>BI.Brain_Inferior_Temporal_Lobe.H3K27ac.149.filt.tagAlign.gz;<br>BI.Brain_Mid_Frontal_Lobe.H3K27ac.112.filt.tagAlign.gz;<br>BI.Brain_Mid_Frontal_Lobe.H3K27ac.149.filt.tagAlign.gz;<br>BI.Brain_Substantia_Nigra.H3K27ac.149.DNA_Lib_1847.filt.tagAlign.gz;<br>BI.Brain_Substantia_Nigra.H3K27ac.149.filt.tagAlign.gz
H3K9ac	BI.Brain_Angular_Gyrus.H3K9ac.112.filt.tagAlign.gz;<br>BI.Brain_Anterior_Caudate.H3K9ac.112.filt.tagAlign.gz;<br>BI.Brain_Cingulate_Gyrus.H3K9ac.112.filt.tagAlign.gz;<br>BI.Brain_Hippocampus_Middle.H3K9ac.112.filt.tagAlign.gz;<br>BI.Brain_Inferior_Temporal_Lobe.H3K9ac.112.filt.tagAlign.gz;<br>BI.Brain_Mid_Frontal_Lobe.H3K9ac.112.filt.tagAlign.gz;<br>BI.Brain_Substantia_Nigra.H3K9ac.112.filt.tagAlign.gz;<br>UCSF-UBC.Fetal_Brain.H3K9ac.HuFNSC-T.filt.tagAlign.gz

H3K4me3	BI.Brain_Angular_Gyrus.H3K4me3.112.filt.tagAlign.gz; BI.Brain_Angular_Gyrus.H3K4me3.149.filt.tagAlign.gz; BI.Brain_Anterior_Caudate.H3K4me3.112.filt.tagAlign.gz; BI.Brain_Anterior_Caudate.H3K4me3.149.filt.tagAlign.gz; BI.Brain_Cingulate_Gyrus.H3K4me3.112.filt.tagAlign.gz; BI.Brain_Cingulate_Gyrus.H3K4me3.149.filt.tagAlign.gz; BI.Brain_Hippocampus_Middle.H3K4me3.112.filt.tagAlign.gz; BI.Brain_Hippocampus_Middle.H3K4me3.149.filt.tagAlign.gz; BI.Brain_Hippocampus_Middle.H3K4me3.150.filt.tagAlign.gz; BI.Brain_Inferior_Temporal_Lobe.H3K4me3.112.filt.tagAlign.gz; BI.Brain_Inferior_Temporal_Lobe.H3K4me3.149.filt.tagAlign.gz; BI.Brain_Mid_Frontal_Lobe.H3K4me3.112.filt.tagAlign.gz; BI.Brain_Mid_Frontal_Lobe.H3K4me3.149.filt.tagAlign.gz; BI.Brain_Substantia_Nigra.H3K4me3.112.filt.tagAlign.gz; BI.Brain_Substantia_Nigra.H3K4me3.149.filt.tagAlign.gz; BI.Fetal_Brain.H3K4me3.UW_H-22510.filt.tagAlign.gz; UCSF-UBC.Brain_Germinal_Matrix.H3K4me3.HuFGM01.filt.tagAlign.gz; UCSF-UBC.Brain_Germinal_Matrix.H3K4me3.HuFGM02.filt.tagAlign.gz; UCSF-UBC.Fetal_Brain.H3K4me3.HuFNSC-T.filt.tagAlign.gz; UCSF-UBC.Fetal_Brain.H3K4me3.HuFNSC01.filt.tagAlign.gz; UCSF-UBC.Fetal_Brain.H3K4me3.HuFNSC02.filt.tagAlign.gz
H3K36me3	BI.Brain_Angular_Gyrus.H3K36me3.112.filt.tagAlign.gz; BI.Brain_Angular_Gyrus.H3K36me3.149.filt.tagAlign.gz; BI.Brain_Anterior_Caudate.H3K36me3.112.filt.tagAlign.gz; BI.Brain_Anterior_Caudate.H3K36me3.149.filt.tagAlign.gz; BI.Brain_Cingulate_Gyrus.H3K36me3.112.filt.tagAlign.gz; BI.Brain_Cingulate_Gyrus.H3K36me3.149.filt.tagAlign.gz; BI.Brain_Hippocampus_Middle.H3K36me3.112.filt.tagAlign.gz; BI.Brain_Hippocampus_Middle.H3K36me3.149.filt.tagAlign.gz; BI.Brain_Hippocampus_Middle.H3K36me3.150.filt.tagAlign.gz; BI.Brain_Inferior_Temporal_Lobe.H3K36me3.112.filt.tagAlign.gz; BI.Brain_Inferior_Temporal_Lobe.H3K36me3.149.filt.tagAlign.gz; BI.Brain_Mid_Frontal_Lobe.H3K36me3.112.filt.tagAlign.gz; BI.Brain_Mid_Frontal_Lobe.H3K36me3.149.filt.tagAlign.gz; BI.Brain_Substantia_Nigra.H3K36me3.112.filt.tagAlign.gz; BI.Brain_Substantia_Nigra.H3K36me3.149.filt.tagAlign.gz; BI.Fetal_Brain.H3K36me3.UW_H-22510.filt.tagAlign.gz; UCSF-UBC.Brain_Germinal_Matrix.H3K36me3.HuFGM01.filt.tagAlign.gz; UCSF-UBC.Brain_Germinal_Matrix.H3K36me3.HuFGM02.filt.tagAlign.gz; UCSF-UBC.Fetal_Brain.H3K36me3.HuFNSC01.filt.tagAlign.gz; UCSF-UBC.Fetal_Brain.H3K36me3.HuFNSC02.filt.tagAlign.gz
H3K27me3	BI.Brain_Angular_Gyrus.H3K27me3.149.filt.tagAlign.gz; BI.Brain_Anterior_Caudate.H3K27me3.112.filt.tagAlign.gz; BI.Brain_Anterior_Caudate.H3K27me3.149.filt.tagAlign.gz; BI.Brain_Cingulate_Gyrus.H3K27me3.149.filt.tagAlign.gz; BI.Brain_Hippocampus_Middle.H3K27me3.112.filt.tagAlign.gz; BI.Brain_Hippocampus_Middle.H3K27me3.149.filt.tagAlign.gz;

	BI.Brain_Hippocampus_Middle.H3K27me3.150.filt.tagAlign.gz; BI.Brain_Inferior_Temporal_Lobe.H3K27me3.112.filt.tagAlign.gz; BI.Brain_Inferior_Temporal_Lobe.H3K27me3.149.filt.tagAlign.gz; BI.Brain_Mid_Frontal_Lobe.H3K27me3.149.filt.tagAlign.gz; BI.Brain_Substantia_Nigra.H3K27me3.112.filt.tagAlign.gz; BI.Brain_Substantia_Nigra.H3K27me3.149.filt.tagAlign.gz; BI.Fetal_Brain.H3K27me3.UW_H-22510.filt.tagAlign.gz; BI.Fetal_Brain.H3K27me3.UW_H22676.filt.tagAlign.gz; UCSF-UBC.Brain_Germinal_Matrix.H3K27me3.HuFGM01.filt.tagAlign.gz; UCSF-UBC.Brain_Germinal_Matrix.H3K27me3.HuFGM02.filt.tagAlign.gz; UCSF-UBC.Fetal_Brain.H3K27me3.HuFNSC-T.filt.tagAlign.gz; UCSF-UBC.Fetal_Brain.H3K27me3.HuFNSC01.filt.tagAlign.gz; UCSF-UBC.Fetal_Brain.H3K27me3.HuFNSC02.filt.tagAlign.gz
H3K9me3	BI.Brain_Angular_Gyrus.H3K9me3.112.filt.tagAlign.gz; BI.Brain_Angular_Gyrus.H3K9me3.149.filt.tagAlign.gz; BI.Brain_Anterior_Caudate.H3K9me3.112.filt.tagAlign.gz; BI.Brain_Anterior_Caudate.H3K9me3.149.filt.tagAlign.gz; BI.Brain_Cingulate_Gyrus.H3K9me3.112.filt.tagAlign.gz; BI.Brain_Cingulate_Gyrus.H3K9me3.149.filt.tagAlign.gz; BI.Brain_Hippocampus_Middle.H3K9me3.112.filt.tagAlign.gz; BI.Brain_Hippocampus_Middle.H3K9me3.149.filt.tagAlign.gz; BI.Brain_Hippocampus_Middle.H3K9me3.150.filt.tagAlign.gz; BI.Brain_Inferior_Temporal_Lobe.H3K9me3.112.filt.tagAlign.gz; BI.Brain_Inferior_Temporal_Lobe.H3K9me3.149.filt.tagAlign.gz; BI.Brain_Mid_Frontal_Lobe.H3K9me3.112.filt.tagAlign.gz; BI.Brain_Mid_Frontal_Lobe.H3K9me3.149.filt.tagAlign.gz; BI.Brain_Substantia_Nigra.H3K9me3.112.filt.tagAlign.gz; BI.Brain_Substantia_Nigra.H3K9me3.149.filt.tagAlign.gz; BI.Fetal_Brain.H3K9me3.UW_H-22510.filt.tagAlign.gz; BI.Fetal_Brain.H3K9me3.UW_H22676.filt.tagAlign.gz; UCSF-UBC.Brain_Germinal_Matrix.H3K9me3.HuFGM01.filt.tagAlign.gz; UCSF-UBC.Brain_Germinal_Matrix.H3K9me3.HuFGM02.filt.tagAlign.gz; UCSF-UBC.Fetal_Brain.H3K9me3.HuFNSC01.filt.tagAlign.gz; UCSF-UBC.Fetal_Brain.H3K9me3.HuFNSC02.filt.tagAlign.gz

Supplementary Table S1. Histone ChIP-seq data in all brain regions from Roadmap Epigenomics used by Deep5hmC for evaluating the prediction performance across four developmental stages in "Forebrain Organoid".

Tissue	Histone mark	ChIP-seq Data Source
Bladder	H3K4me1	UCSD.Bladder.H3K4me1.STL003.filt.tagAlign.gz
	H3K4me3	ENCODE_ENCFF009JNQ_H3K4me3_Bladder.bed
Brain	H3K4me1	BI.Brain_Angular_Gyrus.H3K4me1.112.filt.tagAlign.gz;<br>BI.Brain_Angular_Gyrus.H3K4me1.149.filt.tagAlign.gz;<br>BI.Brain_Anterior_Caudate.H3K4me1.112.filt.tagAlign.gz;<br>BI.Brain_Anterior_Caudate.H3K4me1.149.filt.tagAlign.gz;<br>BI.Brain_Cingulate_Gyrus.H3K4me1.112.filt.tagAlign.gz;<br>BI.Brain_Cingulate_Gyrus.H3K4me1.149.filt.tagAlign.gz;<br>BI.Brain_Hippocampus_Middle.H3K4me1.112.filt.tagAlign.gz;<br>BI.Brain_Hippocampus_Middle.H3K4me1.149.filt.tagAlign.gz;<br>BI.Brain_Hippocampus_Middle.H3K4me1.150.filt.tagAlign.gz;<br>BI.Brain_Inferior_Temporal_Lobe.H3K4me1.112.filt.tagAlign.gz;<br>BI.Brain_Inferior_Temporal_Lobe.H3K4me1.149.filt.tagAlign.gz;<br>BI.Brain_Mid_Frontal_Lobe.H3K4me1.112.filt.tagAlign.gz;<br>BI.Brain_Mid_Frontal_Lobe.H3K4me1.149.filt.tagAlign.gz;<br>BI.Brain_Substantia_Nigra.H3K4me1.112.filt.tagAlign.gz;<br>BI.Brain_Substantia_Nigra.H3K4me1.149.filt.tagAlign.gz;<br>BI.Fetal_Brain.H3K4me1.UW_H22676.filt.tagAlign.gz;<br>UCSF-UBC.Brain_Germinal_Matrix.H3K4me1.HuFGM01.filt.tagAlign.gz;<br>UCSF-UBC.Brain_Germinal_Matrix.H3K4me1.HuFGM02.filt.tagAlign.gz;<br>UCSF-UBC.Fetal_Brain.H3K4me1.HuFNSC01.filt.tagAlign.gz;<br>UCSF-UBC.Fetal_Brain.H3K4me1.HuFNSC02.filt.tagAlign.gz
	H3K4me3	BI.Brain_Angular_Gyrus.H3K4me3.112.filt.tagAlign.gz;<br>BI.Brain_Angular_Gyrus.H3K4me3.149.filt.tagAlign.gz;<br>BI.Brain_Anterior_Caudate.H3K4me3.112.filt.tagAlign.gz;<br>BI.Brain_Anterior_Caudate.H3K4me3.149.filt.tagAlign.gz;<br>BI.Brain_Cingulate_Gyrus.H3K4me3.112.filt.tagAlign.gz;<br>BI.Brain_Cingulate_Gyrus.H3K4me3.149.filt.tagAlign.gz;<br>BI.Brain_Hippocampus_Middle.H3K4me3.112.filt.tagAlign.gz;<br>BI.Brain_Hippocampus_Middle.H3K4me3.149.filt.tagAlign.gz;<br>BI.Brain_Hippocampus_Middle.H3K4me3.150.filt.tagAlign.gz;<br>BI.Brain_Inferior_Temporal_Lobe.H3K4me3.112.filt.tagAlign.gz;<br>BI.Brain_Inferior_Temporal_Lobe.H3K4me3.149.filt.tagAlign.gz;<br>BI.Brain_Mid_Frontal_Lobe.H3K4me3.112.filt.tagAlign.gz;<br>BI.Brain_Mid_Frontal_Lobe.H3K4me3.149.filt.tagAlign.gz;<br>BI.Brain_Substantia_Nigra.H3K4me3.112.filt.tagAlign.gz;<br>BI.Brain_Substantia_Nigra.H3K4me3.149.filt.tagAlign.gz;<br>BI.Fetal_Brain.H3K4me3.UW_H-22510.filt.tagAlign.gz;<br>UCSF-UBC.Brain_Germinal_Matrix.H3K4me3.HuFGM01.filt.tagAlign.gz;<br>UCSF-UBC.Brain_Germinal_Matrix.H3K4me3.HuFGM02.filt.tagAlign.gz;<br>UCSF-UBC.Fetal_Brain.H3K4me3.HuFNSC-T.filt.tagAlign.gz;<br>UCSF-UBC.Fetal_Brain.H3K4me3.HuFNSC01.filt.tagAlign.gz;<br>UCSF-UBC.Fetal_Brain.H3K4me3.HuFNSC02.filt.tagAlign.gz
Breast	H3K4me1	UCSF-<br>UBC.Breast_Fibroblast_Primary_Cells.H3K4me1.RM071.filt.tagAlign.gz;

		UCSF-UBC.Breast_Luminal_Epithelial_Cells.H3K4me1.RM080.filt.tagAlign.gz; UCSF-UBC.Breast_Myoepithelial_Cells.H3K4me1.RM066.filt.tagAlign.gz; UCSF-UBC.Breast_Myoepithelial_Cells.H3K4me1.RM080.filt.tagAlign.gz; UCSF-UBC.Breast_vHMEC.H3K4me1.RM035.HS1994.filt.tagAlign.gz; UCSF-UBC.Breast_vHMEC.H3K4me1.RM035.HS2618.filt.tagAlign.gz
	H3K4me3	UCSF-UBC.Breast_Fibroblast_Primary_Cells.H3K4me3.RM071.filt.tagAlign.gz; UCSF-UBC.Breast_Myoepithelial_Cells.H3K4me3.RM066.filt.tagAlign.gz; UCSF-UBC.Breast_Myoepithelial_Cells.H3K4me3.RM080.filt.tagAlign.gz; UCSF-UBC.Breast_vHMEC.H3K4me3.RM035.HS2615.filt.tagAlign.gz
Heart	H3K4me1	BI.Fetal_Heart.H3K4me1.H-23524.filt.tagAlign.gz; BI.Fetal_Heart.H3K4me1.UW_H23914.filt.tagAlign.gz
	H3K4me3	BI.Fetal_Heart.H3K4me3.UW_H23914.filt.tagAlign.gz
Kidney	H3K4me1	BI.Adult_Kidney.H3K4me1.153.filt.tagAlign.gz; BI.Adult_Kidney.H3K4me1.27.filt.tagAlign.gz; BI.Fetal_Kidney.H3K4me1.UW_H-22676.filt.tagAlign.gz
	H3K4me3	BI.Adult_Kidney.H3K4me3.153.filt.tagAlign.gz; BI.Adult_Kidney.H3K4me3.27.filt.tagAlign.gz; BI.Fetal_Kidney.H3K4me3.UW_H-22676.filt.tagAlign.gz
Liver	H3K4me1	BI.Adult_Liver.H3K4me1.3.filt.tagAlign.gz; BI.Adult_Liver.H3K4me1.4.filt.tagAlign.gz; BI.Adult_Liver.H3K4me1.5.filt.tagAlign.gz; UCSD.Adult_Liver.H3K4me1.STL011.filt.tagAlign.gz
	H3K4me3	BI.Adult_Liver.H3K4me3.3.filt.tagAlign.gz; BI.Adult_Liver.H3K4me3.4.filt.tagAlign.gz; BI.Adult_Liver.H3K4me3.5.filt.tagAlign.gz; UCSD.Adult_Liver.H3K4me3.STL011.filt.tagAlign.gz
Lung	H3K4me1	BI.Fetal_Lung.H3K4me1.UW_H-22727.filt.tagAlign.gz; BI.Fetal_Lung.H3K4me1.UW_H22772.filt.tagAlign.gz; BI.Fetal_Lung.H3K4me1.UW_H23266.filt.tagAlign.gz; UCSD.Lung.H3K4me1.STL001.filt.tagAlign.gz; UCSD.Lung.H3K4me1.STL002.filt.tagAlign.gz
	H3K4me3	BI.Fetal_Lung.H3K4me3.UW_H-22676.filt.tagAlign.gz; BI.Fetal_Lung.H3K4me3.UW_H-22727.filt.tagAlign.gz; UCSD.Lung.H3K4me3.STL002.filt.tagAlign.gz
Marrow	H3K4me1	BI.Bone_Marrow_Derived_Mesenchymal_Stem_Cell_Cultured_Cells.H3K4me1.57.filt.tagAlign.gz; BI.Bone_Marrow_Derived_Mesenchymal_Stem_Cell_Cultured_Cells.H3K4me1.58.filt.tagAlign.gz; BI.Bone_Marrow_Derived_Mesenchymal_Stem_Cell_Cultured_Cells.H3K4me1.59.filt.tagAlign.gz; BI.Bone_Marrow_Derived_Mesenchymal_Stem_Cell_Cultured_Cells.H3K4me1.60.filt.tagAlign.gz; BI.Chondrocytes_from_Bone_Marrow_Derived_Mesenchymal_Stem_Cell_Cultured_Cells.H3K4me1.57.filt.tagAlign.gz; BI.Chondrocytes_from_Bone_Marrow_Derived_Mesenchymal_Stem_Cell_Cultured_Cells.H3K4me1.58.filt.tagAlign.gz; BI.Chondrocytes_from_Bone_Marrow_Derived_Mesenchymal_Stem_Cell_Cultured_Cells.H3K4me1.59.filt.tagAlign.gz;

		BI.Chondrocytes_from_Bone_Marrow_Derived_Mesenchymal_Stem_Cell_Cultured_Cells.H3K4me1.60.filt.tagAlign.gz
	H3K4me3	BI.Bone_Marrow_Derived_Mesenchymal_Stem_Cell_Cultured_Cells.H3K4me3.57.filt.tagAlign.gz; BI.Bone_Marrow_Derived_Mesenchymal_Stem_Cell_Cultured_Cells.H3K4me3.58.filt.tagAlign.gz; BI.Bone_Marrow_Derived_Mesenchymal_Stem_Cell_Cultured_Cells.H3K4me3.59.filt.tagAlign.gz; BI.Bone_Marrow_Derived_Mesenchymal_Stem_Cell_Cultured_Cells.H3K4me3.60.filt.tagAlign.gz; BI.Chondrocytes_from_Bone_Marrow_Derived_Mesenchymal_Stem_Cell_Cultured_Cells.H3K4me3.57.filt.tagAlign.gz; BI.Chondrocytes_from_Bone_Marrow_Derived_Mesenchymal_Stem_Cell_Cultured_Cells.H3K4me3.58.filt.tagAlign.gz; BI.Chondrocytes_from_Bone_Marrow_Derived_Mesenchymal_Stem_Cell_Cultured_Cells.H3K4me3.59.filt.tagAlign.gz; BI.Chondrocytes_from_Bone_Marrow_Derived_Mesenchymal_Stem_Cell_Cultured_Cells.H3K4me3.60.filt.tagAlign.gz
Ovary	H3K4me1	UCSD.Ovary.H3K4me1.STL002.filt.tagAlign.gz
	H3K4me3	UCSD.Ovary.H3K4me3.STL002.filt.tagAlign.gz
Pancreas	H3K4me1	UCSD.Pancreas.H3K4me1.STL002.filt.tagAlign.gz; UCSD.Pancreas.H3K4me1.STL003.filt.tagAlign.gz
	H3K4me3	UCSD.Pancreas.H3K4me3.STL003.filt.tagAlign.gz
Placenta	H3K4me1	UCSF-UBC.Placenta_Amnion.H3K4me1.CTL02.filt.tagAlign.gz; UCSF-UBC.Placenta_Chorion_Smooth.H3K4me1.CTL02.filt.tagAlign.gz; UW.Fetal_Placenta.H3K4me1.H-24996.Histone.DS23027.filt.tagAlign.gz
	H3K4me3	UCSF-UBC.Placenta_Amnion.H3K4me3.CTL02.filt.tagAlign.gz; UCSF-UBC.Placenta_Chorion_Smooth.H3K4me3.CTL02.filt.tagAlign.gz; UW.Fetal_Placenta.H3K4me3.H-24996.Histone.DS23300.filt.tagAlign.gz
Prostate	H3K4me1	ENCODE_ENCFF061CPC_H3K4me1_Prostate.bed; ENCODE_ENCFF099KSH_H3K4me1_Prostate.bed; ENCODE_ENCFF162ACA_H3K4me1_Prostate.bed; ENCODE_ENCFF324PUS_H3K4me1_Prostate.bed
	H3K4me3	ENCODE_ENCFF055CAO_H3K4me3_Prostate.bed; ENCODE_ENCFF369SLA_H3K4me3_Prostate.bed; ENCODE_ENCFF483OJJ_H3K4me3_Prostate.bed; ENCODE_ENCFF854ANC_H3K4me3_Prostate.bed
Colon (Sigmoid)	H3K4me1	UCSD.Sigmoid_Colon.H3K4me1.STL001.filt.tagAlign.gz; UCSD.Sigmoid_Colon.H3K4me1.STL003.filt.tagAlign.gz
	H3K4me3	UCSD.Sigmoid_Colon.H3K4me3.STL001.filt.tagAlign.gz; UCSD.Sigmoid_Colon.H3K4me3.STL003.filt.tagAlign.gz
Skin	H3K4me1	ENCODE_ENCFF797BMX_H3K4me1_Skin.bed; UCSF-UBC.Penis_Foreskin_Fibroblast_Primary_Cells.H3K4me1.skin01.filt.tagAlign.gz; UCSF-UBC.Penis_Foreskin_Fibroblast_Primary_Cells.H3K4me1.skin02.filt.tagAlign.gz;

		UCSF-UBC.Penis_Foreskin_Fibroblast_Primary_Cells.H3K4me1.skin03.filt.tagAlign.gz;<br>UCSF-UBC.Penis_Foreskin_Keratinocyte_Primary_Cells.H3K4me1.skin01.filt.tagAlign.gz;<br>UCSF-UBC.Penis_Foreskin_Keratinocyte_Primary_Cells.H3K4me1.skin02.filt.tagAlign.gz;<br>UCSF-UBC.Penis_Foreskin_Keratinocyte_Primary_Cells.H3K4me1.skin03.filt.tagAlign.gz;<br>UCSF-UBC.Penis_Foreskin_Melanocyte_Primary_Cells.H3K4me1.skin01.filt.tagAlign.gz;<br>UCSF-UBC.Penis_Foreskin_Melanocyte_Primary_Cells.H3K4me1.skin02.filt.tagAlign.gz;<br>UCSF-UBC.Penis_Foreskin_Melanocyte_Primary_Cells.H3K4me1.skin03.filt.tagAlign.gz
	H3K4me3	ENCODE_ENCFF258WJE_H3K4me3_Skin.bed;<br>UCSF-UBC.Penis_Foreskin_Fibroblast_Primary_Cells.H3K4me3.skin01.filt.tagAlign.gz;<br>UCSF-UBC.Penis_Foreskin_Fibroblast_Primary_Cells.H3K4me3.skin02.filt.tagAlign.gz;<br>UCSF-UBC.Penis_Foreskin_Fibroblast_Primary_Cells.H3K4me3.skin03.filt.tagAlign.gz;<br>UCSF-UBC.Penis_Foreskin_Keratinocyte_Primary_Cells.H3K4me3.skin01.filt.tagAlign.gz;<br>UCSF-UBC.Penis_Foreskin_Keratinocyte_Primary_Cells.H3K4me3.skin02.filt.tagAlign.gz;<br>UCSF-UBC.Penis_Foreskin_Keratinocyte_Primary_Cells.H3K4me3.skin03.filt.tagAlign.gz;<br>UCSF-UBC.Penis_Foreskin_Melanocyte_Primary_Cells.H3K4me3.skin01.filt.tagAlign.gz;<br>UCSF-UBC.Penis_Foreskin_Melanocyte_Primary_Cells.H3K4me3.skin02.filt.tagAlign.gz;<br>UCSF-UBC.Penis_Foreskin_Melanocyte_Primary_Cells.H3K4me3.skin03.filt.tagAlign.gz
Stomach	H3K4me1	BI.Stomach_Mucosa.H3K4me1.157.filt.tagAlign.gz;<br>BI.Stomach_Smooth_Muscle.H3K4me1.28.filt.tagAlign.gz;

		UW.Fetal_Stomach.H3K4me1.H-24776.Histone.DS22597.filt.tagAlign.gz
	H3K4me3	BI.Stomach_Mucosa.H3K4me3.157.filt.tagAlign.gz;<br>BI.Stomach_Smooth_Muscle.H3K4me3.161.filt.tagAlign.gz;<br>BI.Stomach_Smooth_Muscle.H3K4me3.28.filt.tagAlign.gz;<br>UW.Fetal_Stomach.H3K4me3.H-24639.Histone.DS22598.filt.tagAlign.gz
Testis	H3K4me1	ENCODE_ENCFF020VQJ_H3K4me1_Testis.bed
	H3K4me3	ENCODE_ENCFF007LNP_H3K4me3_Testis.bed;<br>ENCODE_ENCFF796PVK_H3K4me3_Testis.bed
Colon (Transverse)	H3K4me1	ENCODE_ENCFF002YUH_H3K4me1_Colon_Transverse.bed;<br>ENCODE_ENCFF159ZCY_H3K4me1_Colon_Transverse.bed;<br>ENCODE_ENCFF250ADB_H3K4me1_Colon_Transverse.bed;<br>ENCODE_ENCFF530AJW_H3K4me1_Colon_Transverse.bed;<br>ENCODE_ENCFF638YYL_H3K4me1_Colon_Transverse.bed;<br>ENCODE_ENCFF804UEW_H3K4me1_Colon_Transverse.bed;<br>ENCODE_ENCFF813QQE_H3K4me1_Colon_Transverse.bed;<br>ENCODE_ENCFF996EQE_H3K4me1_Colon_Transverse.bed
	H3K4me3	ENCODE_ENCFF128IEV_H3K4me3_Colon_Transverse.bed;<br>ENCODE_ENCFF197YTB_H3K4me3_Colon_Transverse.bed;<br>ENCODE_ENCFF276GOD_H3K4me3_Colon_Transverse.bed;<br>ENCODE_ENCFF598QBZ_H3K4me3_Colon_Transverse.bed;<br>ENCODE_ENCFF600EPC_H3K4me3_Colon_Transverse.bed;<br>ENCODE_ENCFF614HSP_H3K4me3_Colon_Transverse.bed;<br>ENCODE_ENCFF771GCJ_H3K4me3_Colon_Transverse.bed

Supplementary Table S2. Histone ChIP-seq data (H3K4me1 and H3K4me3) used by Deep5hmC for evaluating the prediction performance across 17 human tissues in "Human Tissues".

Condition	Histone Mark	Gender	ChIP-seq Data Source
Alzheimer's disease	H3K27ac	female	ENCFF167PHR
	H3K4me3	female	ENCFF581GEK
Healthy control	H3K27ac	female	ENCFF372LKU
	H3K4me3	female	ENCFF111DCY

Supplementary Table S3. Histone ChIP-seq data (H3K27ac and H3K4me3) used by Deep5hmC for predicting DhMRs in "Kentucky AD".

DNase-seq Data Source
UW.Fetal_Brain.ChromatinAccessibility_Control.H-22510.DS11872.filt.tagAlign.gz
UW.Fetal_Brain.ChromatinAccessibility_Control.H-22510.DS11877.filt.tagAlign.gz
UW.Fetal_Brain.ChromatinAccessibility_Control.H-22911.DS14464.filt.tagAlign.gz
UW.Fetal_Brain.ChromatinAccessibility_Control.H-23266.DS14717.filt.tagAlign.gz
UW.Fetal_Brain.ChromatinAccessibility_Control.H-23266.DS14718.filt.tagAlign.gz

UW.Fetal_Brain.ChromatinAccessibility_Control.H-23284.DS14803.filt.tagAlign.gz
UW.Fetal_Brain.ChromatinAccessibility_Control.H-23284.DS14815.filt.tagAlign.gz
UW.Fetal_Brain.ChromatinAccessibility_Control.H-23399.DS15453.filt.tagAlign.gz
UW.Fetal_Brain.ChromatinAccessibility_Control.H-23548.DS16302.filt.tagAlign.gz
UW.Fetal_Brain.ChromatinAccessibility_Control.H-24279.DS20221.filt.tagAlign.gz
UW.Fetal_Brain.ChromatinAccessibility_Control.H-24297.DS20226.filt.tagAlign.gz
UW.Fetal_Brain.ChromatinAccessibility_Control.H-24381.DS20231.filt.tagAlign.gz
UW.Fetal_Brain.ChromatinAccessibility_Control.H-24510.DNase.DS20780.filt.tagAlign.gz
UW.Fetal_Brain.ChromatinAccessibility.H-22510.DS11872.filt.tagAlign.gz
UW.Fetal_Brain.ChromatinAccessibility.H-22510.DS11877.filt.tagAlign.gz
UW.Fetal_Brain.ChromatinAccessibility.H-22911.DS14464.filt.tagAlign.gz
UW.Fetal_Brain.ChromatinAccessibility.H-23266.DS14717.filt.tagAlign.gz
UW.Fetal_Brain.ChromatinAccessibility.H-23266.DS14718.filt.tagAlign.gz
UW.Fetal_Brain.ChromatinAccessibility.H-23284.DS14803.filt.tagAlign.gz
UW.Fetal_Brain.ChromatinAccessibility.H-23284.DS14815.filt.tagAlign.gz
UW.Fetal_Brain.ChromatinAccessibility.H-23399.DS15453.filt.tagAlign.gz
UW.Fetal_Brain.ChromatinAccessibility.H-23548.DS16302.filt.tagAlign.gz
UW.Fetal_Brain.ChromatinAccessibility.H-24279.DS20221.filt.tagAlign.gz
UW.Fetal_Brain.ChromatinAccessibility.H-24297.DS20226.filt.tagAlign.gz
UW.Fetal_Brain.ChromatinAccessibility.H-24381.DS20231.filt.tagAlign.gz
UW.Fetal_Brain.ChromatinAccessibility.H-24510.DNase.DS20780.filt.tagAlign.gz

Supplementary Table S4. DNase-seq data in all brain regions from Roadmap Epigenomics used by Deep5hmC for evaluating the prediction performance across four developmental stages in "Forebrain Organoid".

Tissue	DNase-seq Data Source
Bladder	ENCFF499YOU
Brain	UW.Fetal_Brain.ChromatinAccessibility_Control.H-22510.DS11872.filt.tagAlign.gz
	UW.Fetal_Brain.ChromatinAccessibility_Control.H-22510.DS11877.filt.tagAlign.gz
	UW.Fetal_Brain.ChromatinAccessibility_Control.H-22911.DS14464.filt.tagAlign.gz
	UW.Fetal_Brain.ChromatinAccessibility_Control.H-23266.DS14717.filt.tagAlign.gz
	UW.Fetal_Brain.ChromatinAccessibility_Control.H-23266.DS14718.filt.tagAlign.gz
	UW.Fetal_Brain.ChromatinAccessibility_Control.H-23284.DS14803.filt.tagAlign.gz
	UW.Fetal_Brain.ChromatinAccessibility_Control.H-23284.DS14815.filt.tagAlign.gz
	UW.Fetal_Brain.ChromatinAccessibility_Control.H-23399.DS15453.filt.tagAlign.gz
	UW.Fetal_Brain.ChromatinAccessibility_Control.H-23548.DS16302.filt.tagAlign.gz
	UW.Fetal_Brain.ChromatinAccessibility_Control.H-24279.DS20221.filt.tagAlign.gz
	UW.Fetal_Brain.ChromatinAccessibility_Control.H-24297.DS20226.filt.tagAlign.gz
	UW.Fetal_Brain.ChromatinAccessibility_Control.H-24381.DS20231.filt.tagAlign.gz
	UW.Fetal_Brain.ChromatinAccessibility_Control.H-24510.DNase.DS20780.filt.tagAlign.gz
	UW.Fetal_Brain.ChromatinAccessibility.H-22510.DS11872.filt.tagAlign.gz
	UW.Fetal_Brain.ChromatinAccessibility.H-22510.DS11877.filt.tagAlign.gz
	UW.Fetal_Brain.ChromatinAccessibility.H-22911.DS14464.filt.tagAlign.gz
	UW.Fetal_Brain.ChromatinAccessibility.H-23266.DS14717.filt.tagAlign.gz
	UW.Fetal_Brain.ChromatinAccessibility.H-23266.DS14718.filt.tagAlign.gz
	UW.Fetal_Brain.ChromatinAccessibility.H-23284.DS14803.filt.tagAlign.gz
	UW.Fetal_Brain.ChromatinAccessibility.H-23284.DS14815.filt.tagAlign.gz
	UW.Fetal_Brain.ChromatinAccessibility.H-23399.DS15453.filt.tagAlign.gz
	UW.Fetal_Brain.ChromatinAccessibility.H-23548.DS16302.filt.tagAlign.gz
	UW.Fetal_Brain.ChromatinAccessibility.H-24279.DS20221.filt.tagAlign.gz
	UW.Fetal_Brain.ChromatinAccessibility.H-24297.DS20226.filt.tagAlign.gz
	UW.Fetal_Brain.ChromatinAccessibility.H-24381.DS20231.filt.tagAlign.gz
	UW.Fetal_Brain.ChromatinAccessibility.H-24510.DNase.DS20780.filt.tagAlign.gz
Breast	UW.Breast_vHMEC.ChromatinAccessibility_Control.RM035.DS18406.filt.tagAlign.gz
	UW.Breast_vHMEC.ChromatinAccessibility_Control.RM035.DS18438.filt.tagAlign.gz
	UW.Breast_vHMEC.ChromatinAccessibility.RM035.DS18406.filt.tagAlign.gz
	UW.Breast_vHMEC.ChromatinAccessibility.RM035.DS18438.filt.tagAlign.gz
Heart	UW.Fetal_Heart.ChromatinAccessibility_Control.H-22662.DS12531.filt.tagAlign.gz
	UW.Fetal_Heart.ChromatinAccessibility_Control.H-22727.DS12810.filt.tagAlign.gz
	UW.Fetal_Heart.ChromatinAccessibility_Control.H-23468.DS15839.filt.tagAlign.gz
	UW.Fetal_Heart.ChromatinAccessibility_Control.H-23500.DS16018.filt.tagAlign.gz
	UW.Fetal_Heart.ChromatinAccessibility_Control.H-23524.DS16146.filt.tagAlign.gz
	UW.Fetal_Heart.ChromatinAccessibility_Control.H-23589.DS16500.filt.tagAlign.gz

	UW.Fetal_Heart.ChromatinAccessibility_Control.H-23604.DS16582.filt.tagAlign.gz
	UW.Fetal_Heart.ChromatinAccessibility_Control.H-23617.DS16621.filt.tagAlign.gz
	UW.Fetal_Heart.ChromatinAccessibility_Control.H-23663.DS16819.filt.tagAlign.gz
	UW.Fetal_Heart.ChromatinAccessibility_Control.H-23744.DS19431.filt.tagAlign.gz
	UW.Fetal_Heart.ChromatinAccessibility_Control.H-24042.DS19427.filt.tagAlign.gz
	UW.Fetal_Heart.ChromatinAccessibility.H-22662.DS12531.filt.tagAlign.gz
	UW.Fetal_Heart.ChromatinAccessibility.H-22727.DS12810.filt.tagAlign.gz
	UW.Fetal_Heart.ChromatinAccessibility.H-23468.DS15839.filt.tagAlign.gz
	UW.Fetal_Heart.ChromatinAccessibility.H-23500.DS16018.filt.tagAlign.gz
	UW.Fetal_Heart.ChromatinAccessibility.H-23524.DS16146.filt.tagAlign.gz
	UW.Fetal_Heart.ChromatinAccessibility.H-23589.DS16500.filt.tagAlign.gz
	UW.Fetal_Heart.ChromatinAccessibility.H-23604.DS16582.filt.tagAlign.gz
	UW.Fetal_Heart.ChromatinAccessibility.H-23617.DS16621.filt.tagAlign.gz
	UW.Fetal_Heart.ChromatinAccessibility.H-23663.DS16819.filt.tagAlign.gz
	UW.Fetal_Heart.ChromatinAccessibility.H-23744.DS19431.filt.tagAlign.gz
	UW.Fetal_Heart.ChromatinAccessibility.H-24042.DS19427.filt.tagAlign.gz
	UW.Heart.ChromatinAccessibility_Control.STL001.DNase.DS20383.filt.tagAlign.gz
	UW.Heart.ChromatinAccessibility.STL001.DNase.DS20383.filt.tagAlign.gz
Kidney	UW.Fetal_Kidney_Left.ChromatinAccessibility_Control.H-23589.DS16446.filt.tagAlign.gz
	UW.Fetal_Kidney_Left.ChromatinAccessibility_Control.H-23604.DS16579.filt.tagAlign.gz
	UW.Fetal_Kidney_Left.ChromatinAccessibility_Control.H-23640.DS16805.filt.tagAlign.gz
	UW.Fetal_Kidney_Left.ChromatinAccessibility_Control.H-23758.DS17140.filt.tagAlign.gz
	UW.Fetal_Kidney_Left.ChromatinAccessibility_Control.H-24089.DS18466.filt.tagAlign.gz
	UW.Fetal_Kidney_Left.ChromatinAccessibility_Control.H-24568.DNase.DS20920.filt.tagAlign.gz
	UW.Fetal_Kidney_Left.ChromatinAccessibility_Control.H-24582.DNase.DS20953.filt.tagAlign.gz
	UW.Fetal_Kidney_Left.ChromatinAccessibility_Control.H-24626.DNase.DS21344.filt.tagAlign.gz
	UW.Fetal_Kidney_Left.ChromatinAccessibility.H-23589.DS16446.filt.tagAlign.gz
	UW.Fetal_Kidney_Left.ChromatinAccessibility.H-23604.DS16579.filt.tagAlign.gz
	UW.Fetal_Kidney_Left.ChromatinAccessibility.H-23640.DS16805.filt.tagAlign.gz
	UW.Fetal_Kidney_Left.ChromatinAccessibility.H-23758.DS17140.filt.tagAlign.gz
	UW.Fetal_Kidney_Left.ChromatinAccessibility.H-24089.DS18466.filt.tagAlign.gz
	UW.Fetal_Kidney_Left.ChromatinAccessibility.H-24568.DNase.DS20920.filt.tagAlign.gz
	UW.Fetal_Kidney_Left.ChromatinAccessibility.H-24582.DNase.DS20953.filt.tagAlign.gz

	UW.Fetal_Kidney_Left.ChromatinAccessibility.H-24626.DNase.DS21344.filt.tagAlign.gz
	UW.Fetal_Kidney_Right.ChromatinAccessibility_Control.H-23435.DS15651.filt.tagAlign.gz
	UW.Fetal_Kidney_Right.ChromatinAccessibility_Control.H-23589.DS16441.filt.tagAlign.gz
	UW.Fetal_Kidney_Right.ChromatinAccessibility_Control.H-23640.DS16801.filt.tagAlign.gz
	UW.Fetal_Kidney_Right.ChromatinAccessibility_Control.H-23758.DS17144.filt.tagAlign.gz
	UW.Fetal_Kidney_Right.ChromatinAccessibility_Control.H-24089.DS18463.filt.tagAlign.gz
	UW.Fetal_Kidney_Right.ChromatinAccessibility_Control.H-24568.DS20917.filt.tagAlign.gz
	UW.Fetal_Kidney_Right.ChromatinAccessibility_Control.H-24582.DNase.DS20951.filt.tagAlign.gz
	UW.Fetal_Kidney_Right.ChromatinAccessibility_Control.H-24595.DNase.DS21044.filt.tagAlign.gz
	UW.Fetal_Kidney_Right.ChromatinAccessibility_Control.H-24626.DNase.DS21340.filt.tagAlign.gz
	UW.Fetal_Kidney_Right.ChromatinAccessibility.H-23435.DS15651.filt.tagAlign.gz
	UW.Fetal_Kidney_Right.ChromatinAccessibility.H-23589.DS16441.filt.tagAlign.gz
	UW.Fetal_Kidney_Right.ChromatinAccessibility.H-23640.DS16801.filt.tagAlign.gz
	UW.Fetal_Kidney_Right.ChromatinAccessibility.H-23758.DS17144.filt.tagAlign.gz
	UW.Fetal_Kidney_Right.ChromatinAccessibility.H-24089.DS18463.filt.tagAlign.gz
	UW.Fetal_Kidney_Right.ChromatinAccessibility.H-24568.DS20917.filt.tagAlign.gz
	UW.Fetal_Kidney_Right.ChromatinAccessibility.H-24582.DNase.DS20951.filt.tagAlign.gz
	UW.Fetal_Kidney_Right.ChromatinAccessibility.H-24595.DNase.DS21044.filt.tagAlign.gz
	UW.Fetal_Kidney_Right.ChromatinAccessibility.H-24626.DNase.DS21340.filt.tagAlign.gz
	UW.Fetal_Kidney.ChromatinAccessibility_Control.H-22337.DS10986.filt.tagAlign.gz
	UW.Fetal_Kidney.ChromatinAccessibility_Control.H-22676.DS12635.filt.tagAlign.gz
	UW.Fetal_Kidney.ChromatinAccessibility_Control.H-23524.DS16139.filt.tagAlign.gz
	UW.Fetal_Kidney.ChromatinAccessibility_Control.H-23663.DS16837.filt.tagAlign.gz
	UW.Fetal_Kidney.ChromatinAccessibility_Control.H-23855.DS17522.filt.tagAlign.gz
	UW.Fetal_Kidney.ChromatinAccessibility_Control.H-23914.DS17753.filt.tagAlign.gz
	UW.Fetal_Kidney.ChromatinAccessibility_Control.H-24507.DS20564.filt.tagAlign.gz
	UW.Fetal_Kidney.ChromatinAccessibility_Control.H-24510.DNase.DS20786.filt.tagAlign.gz
	UW.Fetal_Kidney.ChromatinAccessibility_Control.H-24584.DS20987.filt.tagAlign.gz
	UW.Fetal_Kidney.ChromatinAccessibility_Control.H-24608.DNase.DS21096.filt.tagAlign.gz
	UW.Fetal_Kidney.ChromatinAccessibility.H-22337.DS10986.filt.tagAlign.gz

	UW.Fetal_Kidney.ChromatinAccessibility.H-22676.DS12635.filt.tagAlign.gz
	UW.Fetal_Kidney.ChromatinAccessibility.H-23524.DS16139.filt.tagAlign.gz
	UW.Fetal_Kidney.ChromatinAccessibility.H-23663.DS16837.filt.tagAlign.gz
	UW.Fetal_Kidney.ChromatinAccessibility.H-23855.DS17522.filt.tagAlign.gz
	UW.Fetal_Kidney.ChromatinAccessibility.H-23914.DS17753.filt.tagAlign.gz
	UW.Fetal_Kidney.ChromatinAccessibility.H-24507.DS20564.filt.tagAlign.gz
	UW.Fetal_Kidney.ChromatinAccessibility.H-24510.DNase.DS20786.filt.tagAlign.gz
	UW.Fetal_Kidney.ChromatinAccessibility.H-24584.DS20987.filt.tagAlign.gz
	UW.Fetal_Kidney.ChromatinAccessibility.H-24608.DNase.DS21096.filt.tagAlign.gz
Liver	ENCFF434AMR
Lung	UW.Fetal_Lung_Left.ChromatinAccessibility_Control.H-23435.DS15637.filt.tagAlign.gz
	UW.Fetal_Lung_Left.ChromatinAccessibility_Control.H-23604.DS16570.filt.tagAlign.gz
	UW.Fetal_Lung_Left.ChromatinAccessibility_Control.H-23640.DS18170.filt.tagAlign.gz
	UW.Fetal_Lung_Left.ChromatinAccessibility_Control.H-23744.DS17105.filt.tagAlign.gz
	UW.Fetal_Lung_Left.ChromatinAccessibility_Control.H-23758.DS17154.filt.tagAlign.gz
	UW.Fetal_Lung_Left.ChromatinAccessibility_Control.H-23833.DS17464.filt.tagAlign.gz
	UW.Fetal_Lung_Left.ChromatinAccessibility_Control.H-23887.DS17674.filt.tagAlign.gz
	UW.Fetal_Lung_Left.ChromatinAccessibility_Control.H-23914.DS17739.filt.tagAlign.gz
	UW.Fetal_Lung_Left.ChromatinAccessibility_Control.H-23964.DS17835.filt.tagAlign.gz
	UW.Fetal_Lung_Left.ChromatinAccessibility_Control.H-24005.DS17959.filt.tagAlign.gz
	UW.Fetal_Lung_Left.ChromatinAccessibility_Control.H-24089.DS18421.filt.tagAlign.gz
	UW.Fetal_Lung_Left.ChromatinAccessibility_Control.H-24111.DS18487.filt.tagAlign.gz
	UW.Fetal_Lung_Left.ChromatinAccessibility_Control.H-24626.DNase.DS21334.filt.tagAlign.gz
	UW.Fetal_Lung_Left.ChromatinAccessibility.H-23435.DS15637.filt.tagAlign.gz
	UW.Fetal_Lung_Left.ChromatinAccessibility.H-23604.DS16570.filt.tagAlign.gz
	UW.Fetal_Lung_Left.ChromatinAccessibility.H-23640.DS18170.filt.tagAlign.gz
	UW.Fetal_Lung_Left.ChromatinAccessibility.H-23744.DS17105.filt.tagAlign.gz
	UW.Fetal_Lung_Left.ChromatinAccessibility.H-23758.DS17154.filt.tagAlign.gz
	UW.Fetal_Lung_Left.ChromatinAccessibility.H-23833.DS17464.filt.tagAlign.gz
	UW.Fetal_Lung_Left.ChromatinAccessibility.H-23887.DS17674.filt.tagAlign.gz
	UW.Fetal_Lung_Left.ChromatinAccessibility.H-23914.DS17739.filt.tagAlign.gz

	UW.Fetal_Lung_Left.ChromatinAccessibility.H-23964.DS17835.filt.tagAlign.gz
	UW.Fetal_Lung_Left.ChromatinAccessibility.H-24005.DS17959.filt.tagAlign.gz
	UW.Fetal_Lung_Left.ChromatinAccessibility.H-24089.DS18421.filt.tagAlign.gz
	UW.Fetal_Lung_Left.ChromatinAccessibility.H-24111.DS18487.filt.tagAlign.gz
	UW.Fetal_Lung_Left.ChromatinAccessibility.H-24626.DNase.DS21334.filt.tagAlign.gz
	UW.Fetal_Lung_Right.ChromatinAccessibility_Control.H-23435.DS15632.filt.tagAlign.gz
	UW.Fetal_Lung_Right.ChromatinAccessibility_Control.H-23604.DS16566.filt.tagAlign.gz
	UW.Fetal_Lung_Right.ChromatinAccessibility_Control.H-23640.DS16790.filt.tagAlign.gz
	UW.Fetal_Lung_Right.ChromatinAccessibility_Control.H-23744.DS17101.filt.tagAlign.gz
	UW.Fetal_Lung_Right.ChromatinAccessibility_Control.H-23758.DS17162.filt.tagAlign.gz
	UW.Fetal_Lung_Right.ChromatinAccessibility_Control.H-23887.DS17670.filt.tagAlign.gz
	UW.Fetal_Lung_Right.ChromatinAccessibility_Control.H-23964.DS17831.filt.tagAlign.gz
	UW.Fetal_Lung_Right.ChromatinAccessibility_Control.H-24005.DS17954.filt.tagAlign.gz
	UW.Fetal_Lung_Right.ChromatinAccessibility_Control.H-24089.DS18418.filt.tagAlign.gz
	UW.Fetal_Lung_Right.ChromatinAccessibility_Control.H-24111.DS18492.filt.tagAlign.gz
	UW.Fetal_Lung_Right.ChromatinAccessibility_Control.H-24626.DNase.DS21328.filt.tagAlign.gz
	UW.Fetal_Lung_Right.ChromatinAccessibility.H-23435.DS15632.filt.tagAlign.gz
	UW.Fetal_Lung_Right.ChromatinAccessibility.H-23604.DS16566.filt.tagAlign.gz
	UW.Fetal_Lung_Right.ChromatinAccessibility.H-23640.DS16790.filt.tagAlign.gz
	UW.Fetal_Lung_Right.ChromatinAccessibility.H-23744.DS17101.filt.tagAlign.gz
	UW.Fetal_Lung_Right.ChromatinAccessibility.H-23758.DS17162.filt.tagAlign.gz
	UW.Fetal_Lung_Right.ChromatinAccessibility.H-23887.DS17670.filt.tagAlign.gz
	UW.Fetal_Lung_Right.ChromatinAccessibility.H-23964.DS17831.filt.tagAlign.gz
	UW.Fetal_Lung_Right.ChromatinAccessibility.H-24005.DS17954.filt.tagAlign.gz
	UW.Fetal_Lung_Right.ChromatinAccessibility.H-24089.DS18418.filt.tagAlign.gz
	UW.Fetal_Lung_Right.ChromatinAccessibility.H-24111.DS18492.filt.tagAlign.gz
	UW.Fetal_Lung_Right.ChromatinAccessibility.H-24626.DNase.DS21328.filt.tagAlign.gz
	UW.Fetal_Lung.ChromatinAccessibility_Control.H-22676.DS12646.filt.tagAlign.gz
	UW.Fetal_Lung.ChromatinAccessibility_Control.H-22727.DS12817.filt.tagAlign.gz
	UW.Fetal_Lung.ChromatinAccessibility_Control.H-22934B.DS13507.filt.tagAlign.gz
	UW.Fetal_Lung.ChromatinAccessibility_Control.H-23090.DS13985.filt.tagAlign.gz
	UW.Fetal_Lung.ChromatinAccessibility_Control.H-23247.DS14666.filt.tagAlign.gz

	UW.Fetal_Lung.ChromatinAccessibility_Control.H-23266.DS14724.filt.tagAlign.gz
	UW.Fetal_Lung.ChromatinAccessibility_Control.H-23266.DS14751.filt.tagAlign.gz
	UW.Fetal_Lung.ChromatinAccessibility_Control.H-23284.DS14809.filt.tagAlign.gz
	UW.Fetal_Lung.ChromatinAccessibility_Control.H-23284.DS14820.filt.tagAlign.gz
	UW.Fetal_Lung.ChromatinAccessibility_Control.H-23365.DS15227.filt.tagAlign.gz
	UW.Fetal_Lung.ChromatinAccessibility_Control.H-23399.DS15461.filt.tagAlign.gz
	UW.Fetal_Lung.ChromatinAccessibility_Control.H-23419.DS15573.filt.tagAlign.gz
	UW.Fetal_Lung.ChromatinAccessibility.H-22676.DS12646.filt.tagAlign.gz
	UW.Fetal_Lung.ChromatinAccessibility.H-22727.DS12817.filt.tagAlign.gz
	UW.Fetal_Lung.ChromatinAccessibility.H-22934B.DS13507.filt.tagAlign.gz
	UW.Fetal_Lung.ChromatinAccessibility.H-23090.DS13985.filt.tagAlign.gz
	UW.Fetal_Lung.ChromatinAccessibility.H-23247.DS14666.filt.tagAlign.gz
	UW.Fetal_Lung.ChromatinAccessibility.H-23266.DS14724.filt.tagAlign.gz
	UW.Fetal_Lung.ChromatinAccessibility.H-23266.DS14751.filt.tagAlign.gz
	UW.Fetal_Lung.ChromatinAccessibility.H-23284.DS14809.filt.tagAlign.gz
	UW.Fetal_Lung.ChromatinAccessibility.H-23284.DS14820.filt.tagAlign.gz
	UW.Fetal_Lung.ChromatinAccessibility.H-23365.DS15227.filt.tagAlign.gz
	UW.Fetal_Lung.ChromatinAccessibility.H-23399.DS15461.filt.tagAlign.gz
	UW.Fetal_Lung.ChromatinAccessibility.H-23419.DS15573.filt.tagAlign.gz
Marrow	ENCFF323PMM
Ovary	UW.Fetal_Ovary.ChromatinAccessibility_Control.H23758_H23604.DNase.DS17445.filt.tagAlign.gz
	UW.Fetal_Ovary.ChromatinAccessibility.H23758_H23604.DNase.DS17445.filt.tagAlign.gz
	UW.Ovary.ChromatinAccessibility_Control.STL002.DNase.DS20827.filt.tagAlign.gz
	UW.Ovary.ChromatinAccessibility.STL002.DNase.DS20827.filt.tagAlign.gz
Pancreas	UW.Pancreas.ChromatinAccessibility_Control.STL002.DNase.DS20842.filt.tagAlign.gz
	UW.Pancreas.ChromatinAccessibility_Control.STL003.DNase.DS20753.filt.tagAlign.gz
	UW.Pancreas.ChromatinAccessibility.STL002.DNase.DS20842.filt.tagAlign.gz
	UW.Pancreas.ChromatinAccessibility.STL003.DNase.DS20753.filt.tagAlign.gz
Placenta	UW.Fetal_Placenta.ChromatinAccessibility_Control.H-23887.DS17639.filt.tagAlign.gz
	UW.Fetal_Placenta.ChromatinAccessibility_Control.H-23914.DS17744.filt.tagAlign.gz
	UW.Fetal_Placenta.ChromatinAccessibility_Control.H-24272.DS19391.filt.tagAlign.gz
	UW.Fetal_Placenta.ChromatinAccessibility_Control.H-24409.DS20346.filt.tagAlign.gz
	UW.Fetal_Placenta.ChromatinAccessibility_Control.H-24510.DNase.DS20793.filt.tagAlign.gz
	UW.Fetal_Placenta.ChromatinAccessibility_Control.H-24608.DNase.DS21111.filt.tagAlign.gz
	UW.Fetal_Placenta.ChromatinAccessibility.H-23887.DS17639.filt.tagAlign.gz
	UW.Fetal_Placenta.ChromatinAccessibility.H-23914.DS17744.filt.tagAlign.gz

	UW.Fetal_Placenta.ChromatinAccessibility.H-24272.DS19391.filt.tagAlign.gz
	UW.Fetal_Placenta.ChromatinAccessibility.H-24409.DS20346.filt.tagAlign.gz
	UW.Fetal_Placenta.ChromatinAccessibility.H-24510.DNase.DS20793.filt.tagAlign.gz
	UW.Fetal_Placenta.ChromatinAccessibility.H-24608.DNase.DS21111.filt.tagAlign.gz
Prostate	ENCFF521VPL<br>ENCFF653YLG
Colon (Sigmoid)	ENCFF540ZBW
Skin	UW.Fetal_Skin.ChromatinAccessibility_Control.H-22337.DS10987.filt.tagAlign.gz
	UW.Fetal_Skin.ChromatinAccessibility.H-22337.DS10987.filt.tagAlign.gz
Stomach	UW.Fetal_Stomach.ChromatinAccessibility_Control.H-23589.DS16530.filt.tagAlign.gz
	UW.Fetal_Stomach.ChromatinAccessibility_Control.H-23758.DS17172.filt.tagAlign.gz
	UW.Fetal_Stomach.ChromatinAccessibility_Control.H-23769.DS17325.filt.tagAlign.gz
	UW.Fetal_Stomach.ChromatinAccessibility_Control.H-23887.DS17659.filt.tagAlign.gz
	UW.Fetal_Stomach.ChromatinAccessibility_Control.H-23914.DS17750.filt.tagAlign.gz
	UW.Fetal_Stomach.ChromatinAccessibility_Control.H-23964.DS17878.filt.tagAlign.gz
	UW.Fetal_Stomach.ChromatinAccessibility_Control.H-24005.DS17963.filt.tagAlign.gz
	UW.Fetal_Stomach.ChromatinAccessibility_Control.H-24078.DS18389.filt.tagAlign.gz
	UW.Fetal_Stomach.ChromatinAccessibility_Control.H-24125.DS18821.filt.tagAlign.gz
	UW.Fetal_Stomach.ChromatinAccessibility_Control.H-24342.DS19933.filt.tagAlign.gz
	UW.Fetal_Stomach.ChromatinAccessibility_Control.H-24365.DNase.DS20079.filt.tagAlign.gz
	UW.Fetal_Stomach.ChromatinAccessibility_Control.H-24401.DS20349.filt.tagAlign.gz
	UW.Fetal_Stomach.ChromatinAccessibility_Control.H-24507.DS20546.filt.tagAlign.gz
	UW.Fetal_Stomach.ChromatinAccessibility_Control.H-24510.DNase.DS20791.filt.tagAlign.gz
	UW.Fetal_Stomach.ChromatinAccessibility.H-23589.DS16530.filt.tagAlign.gz
	UW.Fetal_Stomach.ChromatinAccessibility.H-23758.DS17172.filt.tagAlign.gz
	UW.Fetal_Stomach.ChromatinAccessibility.H-23769.DS17325.filt.tagAlign.gz
	UW.Fetal_Stomach.ChromatinAccessibility.H-23887.DS17659.filt.tagAlign.gz
	UW.Fetal_Stomach.ChromatinAccessibility.H-23914.DS17750.filt.tagAlign.gz
	UW.Fetal_Stomach.ChromatinAccessibility.H-23964.DS17878.filt.tagAlign.gz
	UW.Fetal_Stomach.ChromatinAccessibility.H-24005.DS17963.filt.tagAlign.gz
	UW.Fetal_Stomach.ChromatinAccessibility.H-24078.DS18389.filt.tagAlign.gz
	UW.Fetal_Stomach.ChromatinAccessibility.H-24125.DS18821.filt.tagAlign.gz
	UW.Fetal_Stomach.ChromatinAccessibility.H-24342.DS19933.filt.tagAlign.gz
	UW.Fetal_Stomach.ChromatinAccessibility.H-24365.DNase.DS20079.filt.tagAlign.gz
	UW.Fetal_Stomach.ChromatinAccessibility.H-24401.DS20349.filt.tagAlign.gz
	UW.Fetal_Stomach.ChromatinAccessibility.H-24507.DS20546.filt.tagAlign.gz
	UW.Fetal_Stomach.ChromatinAccessibility.H-24510.DNase.DS20791.filt.tagAlign.gz

Testis	ENCFF066TTB
Colon (Transvers e)	ENCFF629OKO

Supplementary Table S5. DNase-seq ChIP-seq data in all brain regions from Roadmap Epigenomics used by Deep5hmC for evaluating the prediction performance across 17 human tissues in "Human Tissues".

Data	Tissue	Number of Positive Peaks
Brain Organoid	EB	64458
	D56	56036
	D84	81032
	D112	81050
Human Tissues	Bladder	98256
	Brain	29079
	Breast	46100
	Heart	75606
	Kidney	75959
	Liver	78819
	Lung	51466
	Marrow	12596
	Ovary	116948
	Pancreas	81555
	Placenta	137488
	Prostate	75271
	Colon (Sigmoid)	25428
	Skin	69322
	Stomach	28270
	Testis	25142
	Colon (Transverse)	32460
Kentucky AD	-	4330

Supplementary Table S6. Summary of sample size for "Brain Organoid", "Human Tissues" and "Kentucky AD".

Histone Mark	ChIP-seq Data Source
H3K4me1	BI.Brain_Angular_Gyrus.H3K4me1.112.filt.tagAlign.gz
H3K27ac	BI.Brain_Angular_Gyrus.H3K27ac.112.filt.tagAlign.gz
H3K9ac	BI.Brain_Angular_Gyrus.H3K9ac.112.filt.tagAlign.gz
H3K4me3	BI.Brain_Angular_Gyrus.H3K4me3.112.filt.tagAlign.gz
H3K36me3	BI.Brain_Angular_Gyrus.H3K36me3.112.filt.tagAlign.gz
H3K27me3	BI.Brain_Angular_Gyrus.H3K27me3.149.filt.tagAlign.gz
H3K9me3	BI.Brain_Angular_Gyrus.H3K9me3.112.filt.tagAlign.gz

Supplementary Table S7. ChIP-seq data related "Brain Angular Gyrus" from Roadmap Epigenomics for exploring the distribution pattern of histone modification around 5hmC peaks in EB stage from "Forebrain Organoid".
